# *In Vivo* Functions of CPSF6 for HIV-1 as Revealed by HIV-1 Capsid Evolution in HLA-B27-Positive Subjects

**DOI:** 10.1371/journal.ppat.1003868

**Published:** 2014-01-09

**Authors:** Matthew S. Henning, Brittany N. Dubose, Mallori J. Burse, Christopher Aiken, Masahiro Yamashita

**Affiliations:** 1 Aaron Diamond AIDS Research Center, New York, New York, United States of America; 2 Department of Pathology, Microbiology and Immunology, Vanderbilt University School of Medicine, Nashville, Tennessee, United States of America; Universitätklinikum Heidelberg, Germany

## Abstract

The host protein CPSF6 possesses a domain that can interact with the HIV-1 capsid (CA) protein. CPSF6 has been implicated in regulating HIV-1 nuclear entry. However, its functional significance for HIV-1 replication has yet to be firmly established. Here we provide evidence for two divergent functions of CPSF6 for HIV-1 replication *in vivo*. We demonstrate that endogenous CPSF6 exerts an inhibitory effect on naturally occurring HIV-1 variants in individuals carrying the HLA-B27 allele. Conversely, we find a strong selective pressure in these individuals to preserve CPSF6 binding, while escaping from the restrictive activity by CPSF6. This active maintenance of CPSF6 binding during HIV-1 CA evolution *in vivo* contrasts with the *in vitro* viral evolution, which can reduce CPSF6 binding to evade from CPSF6-mediated restriction. Thus, these observations argue for a beneficial role of CPSF6 for HIV-1 *in vivo*. CPSF6-mediated restriction renders HIV-1 less dependent or independent from TNPO3, RanBP2 and Nup153, host factors implicated in HIV-1 nuclear entry. However, viral evolution that maintains CPSF6 binding in HLA-B27+ subjects invariably restores the ability to utilize these host factors, which may be the major selective pressure for CPSF6 binding *in vivo*. Our study uncovers two opposing CA-dependent functions of CPSF6 in HIV-1 replication *in vivo*; however, the benefit for binding CPSF6 appears to outweigh the cost, providing support for a vital function of CPSF6 during HIV-1 replication *in vivo*.

## Author Summary

The viral capsid (CA) protein of HIV-1 determines both the ability to infect non-dividing cells and the utilization of host factors implicated in nuclear entry. Understanding how CA controls these two properties is critical. CPSF6, a CA-interacting host protein, may be important for these properties but its precise role remains unclear. Here we provide direct evidence for the involvement of endogenous CPSF6 during HIV-1 infection. We found that CPSF6 blocks CA mutants that are impaired for infection of non-dividing cells. This CPSF6-mediated inhibition also targets early escape variants that arise in HIV-1 infected HLA-B27+ patients. Moreover, this CPSF6-mediated inhibition, together with robust CTL response, appears to be critical for viral suppression, because viruses derived after late viral breakthrough in these individuals were no longer sensitive to the antiviral activity of CPSF6. However, we also report indirect evidence for a potentially beneficial role for CPSF6 in HIV-1 replication, because escape from this inhibition *in vivo* was paradoxically accompanied by a strict preservation of the CPSF6 binding pocket. These results highlight the unique characteristics of the HIV-CPSF6 interactions in which CPSF6 can be either beneficial or detrimental for viral replication in a CA-specific manner.

## Introduction

An essential part of the HIV-1 lifecycle is the transfer of its genetic material from the cytoplasm into the nucleus for subsequent integration into the host genome. In actively proliferating cells, breakdown of the nuclear membrane during mitosis ensures viral access to the host chromosomes. However, HIV-1 and other lentiviruses share the ability to efficiently infect non-dividing cells [1]–[3]. This necessitates a mechanism of hijacking the cellular transport machinery in order for HIV-1 to cross the intact nuclear envelope through nuclear pores [4], [5]. Understanding the mechanism of HIV-1 nuclear entry is crucial [6], [7], as this is the property that enables HIV-1 to infect such critical target cell types *in vivo* as resting or partially activated CD4+ T cells [8], [9] as well as tissue macrophages [10].

Comparative studies utilizing HIV-1 and murine leukemia virus (MLV), a virus unable to efficiently infect non-dividing cells, demonstrated that the viral capsid (CA) protein is the major determinant for HIV-1 infection of non-dividing cells [11]. Since MLV is blocked in non-dividing cells [12], [13] at nuclear entry [14], HIV-1 must be equipped with a CA-dependent mechanism to utilize the host nuclear transport machinery to infect non-dividing cells. Indeed, there is mounting evidence pointing to the role of CA in HIV-1 nuclear entry [11], [15]–[19]. Therefore, one major question is how CA orchestrates interactions between pre-integration complexes (PICs) and host cellular machinery to promote HIV-1 nuclear entry.

Genome-wide siRNA screenings revealed a number of potential cellular factors that could affect HIV-1 nuclear import [20]–[22]. Among these host molecules, transportin 3 (TNPO3), RanBP2 and Nup153 are of particular interest for the following reasons: 1) Knockdown of these molecules blocks HIV-1 infection after reverse transcription but before integration [20], [21], [23]. 2) These molecules are required by HIV-1 but not by MLV [20], [21], [23]. 3) Most importantly, HIV-1 usage of these molecules is determined by CA [17]–[19], [24]–[26]. These findings provide a CA-dependent link between HIV-1 infection of non-dividing cells and host factors exploited by HIV-1 to promote nuclear entry.

An unresolved question is how CA mediates HIV-1 utilization of these host factors (TNPO3, RanBP2 and Nup153). All three proteins can bind to CA [19], [27]–[30]; however, their binding to CA does not perfectly correlate with HIV-1 dependence on these host factors [31]. This suggests the presence of an upstream molecule(s) that can interact with CA to determine the nuclear entry pathway taken by the PIC. One such molecule is cyclophilin A (CypA) because it directly binds to incoming viral capsids [32], [33] and modulates HIV-1 utilization of TNPO3, RanBP2 and Nup153 [19], [26], [34]. CPSF6, which also carries a CA-binding domain, is another molecule that may be important in regulating HIV-1 nuclear entry. In fact, CPSF6 binding to CA more strongly correlates with TNPO3 utilization of HIV-1 than CypA binding [18], [35], [36]. Thus, one attractive hypothesis proposed by Price *et al* is that CPSF6 enables HIV-1 to utilize host factors [36], such as TNPO3, RanBP2 and Nup153, thus setting the course of HIV-1 nuclear entry. However, the precise role of CPSF6 in HIV-1 replication and its physiological relevance have yet to be firmly established, partly because: 1) There is no experimental evidence for the binding of CA to endogenous CPSF6. 2) CPSF6 knockdown has little effect on wild-type (WT) HIV-1 infection *in vitro* [18]. 3) Finally, TNPO3 depletion may keep CPSF6 in the cytoplasm, consequently creating an artificial situation where HIV-1 is effectively blocked [35], [37]. Taken together, it is controversial whether CPSF6 has any direct role in HIV-1 infection.

As mentioned, HIV-1 has an exceptional ability to infect non-dividing cells as efficiently as dividing cells. However, previous studies discovered unusual HIV-1 CA mutants that lost this phenotype and became dependent on cell cycle progression for their infection [38], [39]. One key observation is that most of such cell cycle-dependent CA mutants are blocked by CypA [39]–[41]. The mechanism by which these mutants become sensitive to an inhibitory function of CypA has been controversial, but one report hinted a co-factor that acts together with CypA in thwarting infection by these CA mutants [42]. Here we provide evidence that endogenous CPSF6 acts together with CypA to exert a detrimental effect on such cell cycle-dependent HIV-1 variants, which include naturally occurring HIV-1 CTL escape mutants in individuals carrying HLA-B27 [43]. HIV-1 can escape from CPSF6-mediated restriction by changing or eliminating CPSF6 binding during *in vitro* viral evolution. Surprisingly, the escape pathway *in vivo* is entirely different from *in vitro*, because *in vivo* evolution recurrently selects for viruses that strictly maintain CPSF6 binding, suggesting an opposing, beneficial role of CPSF6 for HIV-1 replication *in vivo*. CPSF6 diminishes the ability of restricted viruses to utilize TNPO3, RanBP2 and Nup153. However, *in vivo* evolution restores this ability. Thus, the preferential selection of CPSF6-dependent viruses *in vivo* may be beneficial by fostering the utilization of HIV-1 cofactors involved in nuclear entry.

## Results

### Endogenous CPSF6 restricts cell cycle-dependent HIV-1 CA mutants

**Figure 1:**
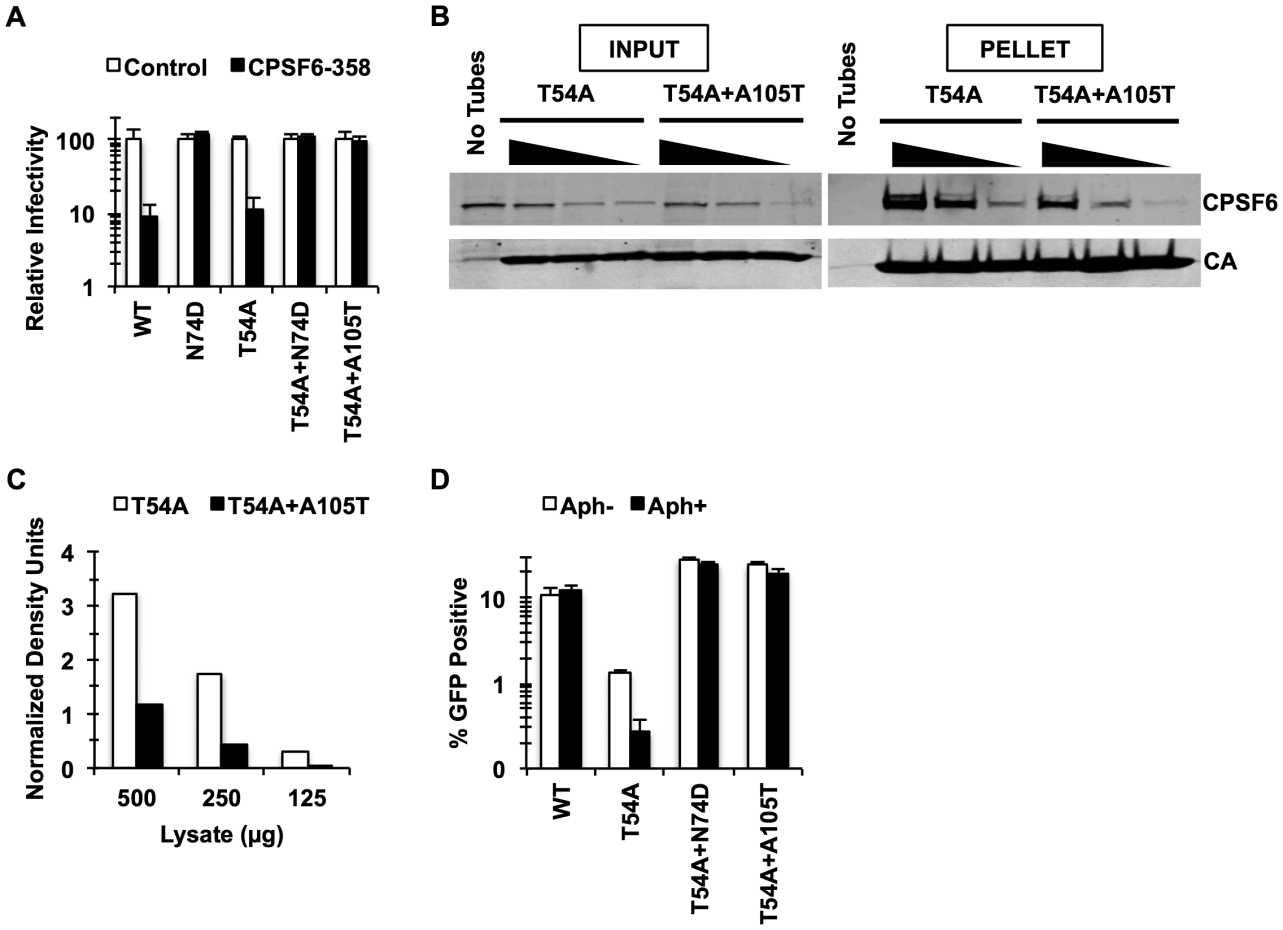
Mutations that reduce CA binding to CPSF6 rescue T54A. (A) HeLa cells stably transduced with the control LPCX vector or one overexpressing CPSF6-358 were infected with VSV-G-pseudotyped GFP reporter viruses. Infectivity of T54A was significantly lower in CPSF6-358-expressing cells than in control cells (p<0.001). (B) T54A and T54A-A105T CA tubes (5 µM) were incubated with 500, 250, or 125 µg of HeLa cell extracts for 1 hr with gentle mixing. The tubes were pelleted, and proteins analyzed by non-reducing SDS-PAGE and immunoblotting for CPSF6 and CA. Input represents 10% of initial reaction. The results are representative of three independent experiments. (C) Quantification of CPSF6 association with CA tubes relative to amount of pelleted CA. (D) HeLa cells were infected with RT-normalized VSV-G-pseudotyped GFP reporter viruses in the presence or absence of aphidicolin (Aph). Reduction of T54A infectivity in aphidicolin-treated cells was statistically significant (p<0.02???). Results are one representative experiment of at least three experiments. Standard deviations of a single triplicate experiment are indicated with the error bars (A and D).

In the present study, we utilized CA mutations localized in the defined CPSF6-binding pocket to explore the function of endogenous CPSF6 for HIV-1 infection. We noticed that A105T, one such CA mutation within the CPSF6-binding pocket [36], was identified as a compensatory mutation to rescue the T54A mutant [44]. The T54A mutant is defective even in proliferating cells; however, as one of the cell cycle-dependent CA mutants, its infection is more severely impaired in non-dividing cells [44]. This observation suggested to us that CPSF6 binding may affect infection of non-dividing cells and that if A105T altered this binding it would rescue cell cycle-dependent CA mutants. To determine whether A105T indeed prevents CA from binding to CPSF6, we first used a restriction assay based on a fragment of CPSF6, called CPSF6-358. In this assay, restriction of infection occurs when viral capsids are recognized by CPSF6-358 [18]. While the T54A CA mutant was as sensitive to CPSF6-358 restriction as WT CA, we find that the introduction of A105T in the T54A CA (**Fig. 1A**), as well as in WT CA (data not shown), rescues infectivity (p<0.01), suggesting that this A105T mutation prevents binding of CPSF6-358. Consistent with this, virions carrying the A105T mutation were unable to abrogate CPSF6-358 restriction when increasing amounts of abrogating virions were used to infect CPSF6-358 expressing cells that were co-infected with the tester WT virus (**Fig. S1**). Finally, to directly test whether A105T inhibits CPSF6 binding, we carried out an *in vitro* binding assay using recombinant HIV-1 CA tubular assemblies. Incubation of the T54A CA tubes with HeLa cell lysates followed by low-speed centrifugation resulted in cosedimentation of endogenous CPSF6 (**Fig. 1B**) at a level comparable to the WT CA tubes (**Fig. S2**). By contrast, T54A+A105T CA tubes brought down one-third of the quantity of CPSF6 as the T54A CA tubes (**Fig. 1C**), indicating that the A105T substitution reduces the association of CPSF6 with the HIV-1 capsid. These findings provide both genetic and biochemical evidence that the A105T substitution reduces the binding of CA to CPSF6.

To confirm that blocking CPSF6 binding to the HIV-1 capsid rescues infection by T54A, we introduced N74D, a mutation known to prevent CPSF6 binding [18], into the T54A mutant virus. Similar to A105T, N74D allowed T54A to escape from inhibition by CPSF6-358 (**Fig. 1A**) and reduced binding of CA tubes to CPSF6 *in vitro* (**Fig. S2**) Moreover, N74D acted similarly to A105T as reported by Qi et al. [39] with respect to its ability to restore T54A infectivity in aphidicolin-arrested HeLa cells (**Fig. 1D**) and infection of actively proliferating HeLa cells (**Fig. 1D**).

**Figure 2:**
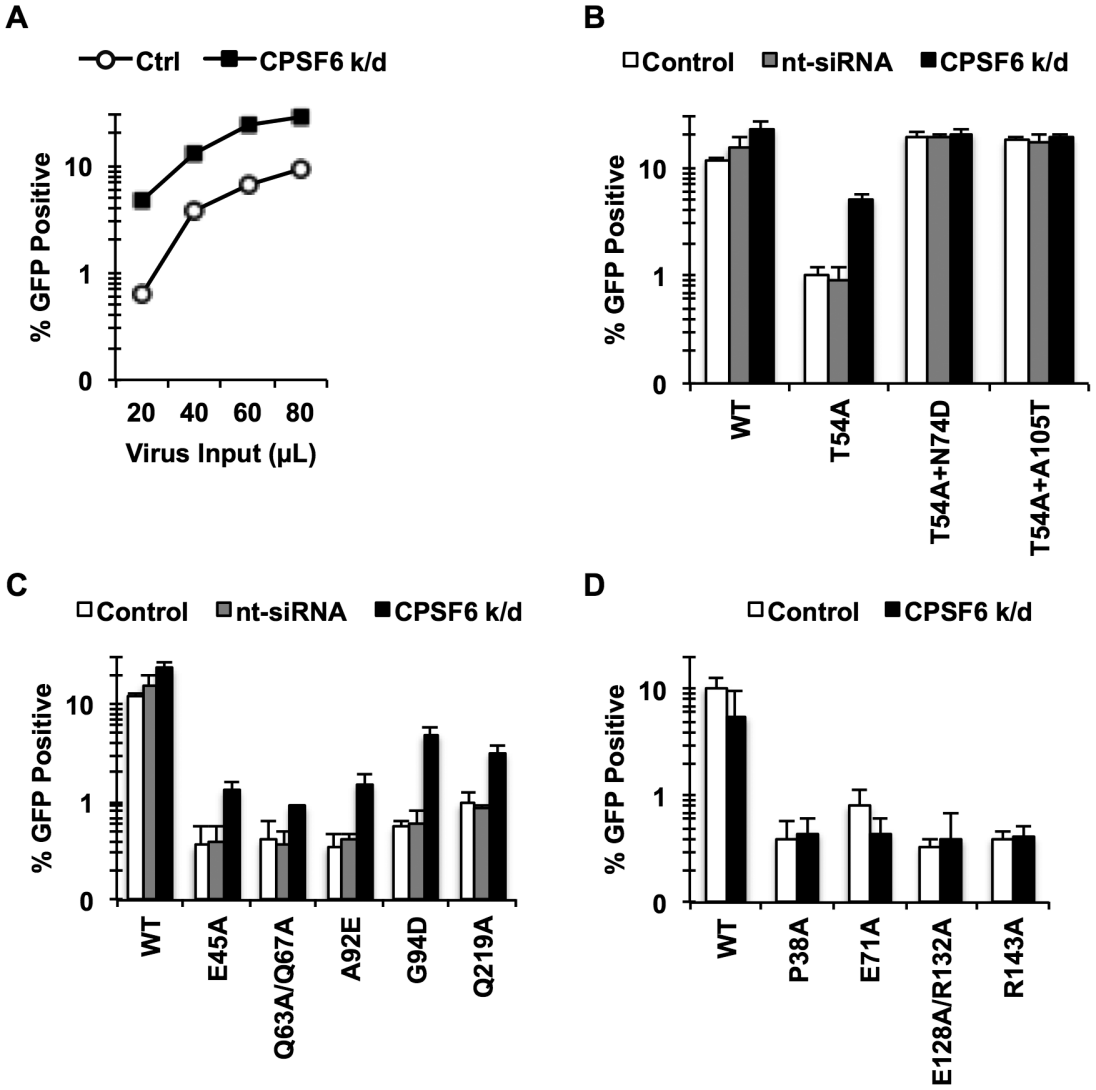
Endogenous CPSF6 restricts cell cycle-dependent HIV-1 CA mutants. (A, B) HeLa cells transfected with either non-targeting siRNA or siRNA targeting CPSF6 were infected with RT-normalized VSV-G-pseudotyped GFP reporter viruses. Increase in viral infectivity of T54A by CPSF6 depletion was statistically significant (B; p<0.002) and different from that of T54A+N74D or T54A+A105T (p<0.001). (C) HeLa cells transfected with either non-targeting siRNA or siRNA targeting CPSF6 were infected with VSV-G-pseudotyped GFP carrying CA mutants that lost the cell cycle independence so that 0.5%-1% cells were infected with the viruses two days after infection. (D) An experiment similar to that described in C was carried out, except that all these CA mutants still maintain the cell cycle independence. Results are one representative experiment of at least two experiments. Standard deviations of a single triplicate experiment are indicated with the error bars.

These observations suggested that mutations in the CPSF6-binding pocket restore T54A infectivity by preventing the binding of CA to CPSF6. As T54A is also inhibited for infection of growth-arrested HeLa cells, we hypothesized that endogenous CPSF6 contributes to inhibition of cell cycle-dependent HIV-1 CA mutants. To test this, we asked whether depleting endogenous CPSF6 would restore infectivity of the T54A mutant virus. CPSF6 depletion by siRNA rendered cells more permissive to infection by T54A (**Fig. 2A** **,** **Fig. 2B** **; p<0.002**). We confirmed this observation by including non-targeting siRNA as controls (**Fig. 2B**) as well as with another CPSF6-targeting siRNA (data not shown). In contrast, the two double mutants (T54A+N74D and T54A+A105T) were not enhanced further by knockdown of CPSF6 (**Fig. 2B**). Moreover, the wild type virus exhibited only modest increase in infectivity upon CPSF6 depletion. Thus, CPSF6 appears to specifically inhibit infection by the HIV-1 T54A mutant.

To determine whether the sensitivity to CPSF6-mediated inhibition is specific to CA mutants that lost the ability to infect non-dividing cells, we examined cell cycle-dependent and –independent CA mutants for their response to CPSF6 knockdown. We depleted endogenous CPSF6 and infected the cells either with CA mutants that become dependent on cell division (E45A, Q63A/Q67A, A92E, G94D, and Q219A) or those that are impaired for infection but maintain cell cycle independence for infection (P38A, E71A, E128A/R132A and R143A) [38]. Infectivity of all of the tested cell cycle-dependent CA mutants was increased upon CPSF6 depletion to a statistically significant extent (**Fig. 2C** **and Fig. S3**). In contrast, infection by none of the cell cycle-independent CA mutants was affected by CPSF6 depletion (**Fig. 2D**). Thus, CPSF6 appears to selectively inhibit cell cycle-dependent CA mutants.

### CPSF6 acts with CypA to suppress early CTL escape variants of HIV-1 in HLA-B27+ subjects

**Figure 3:**
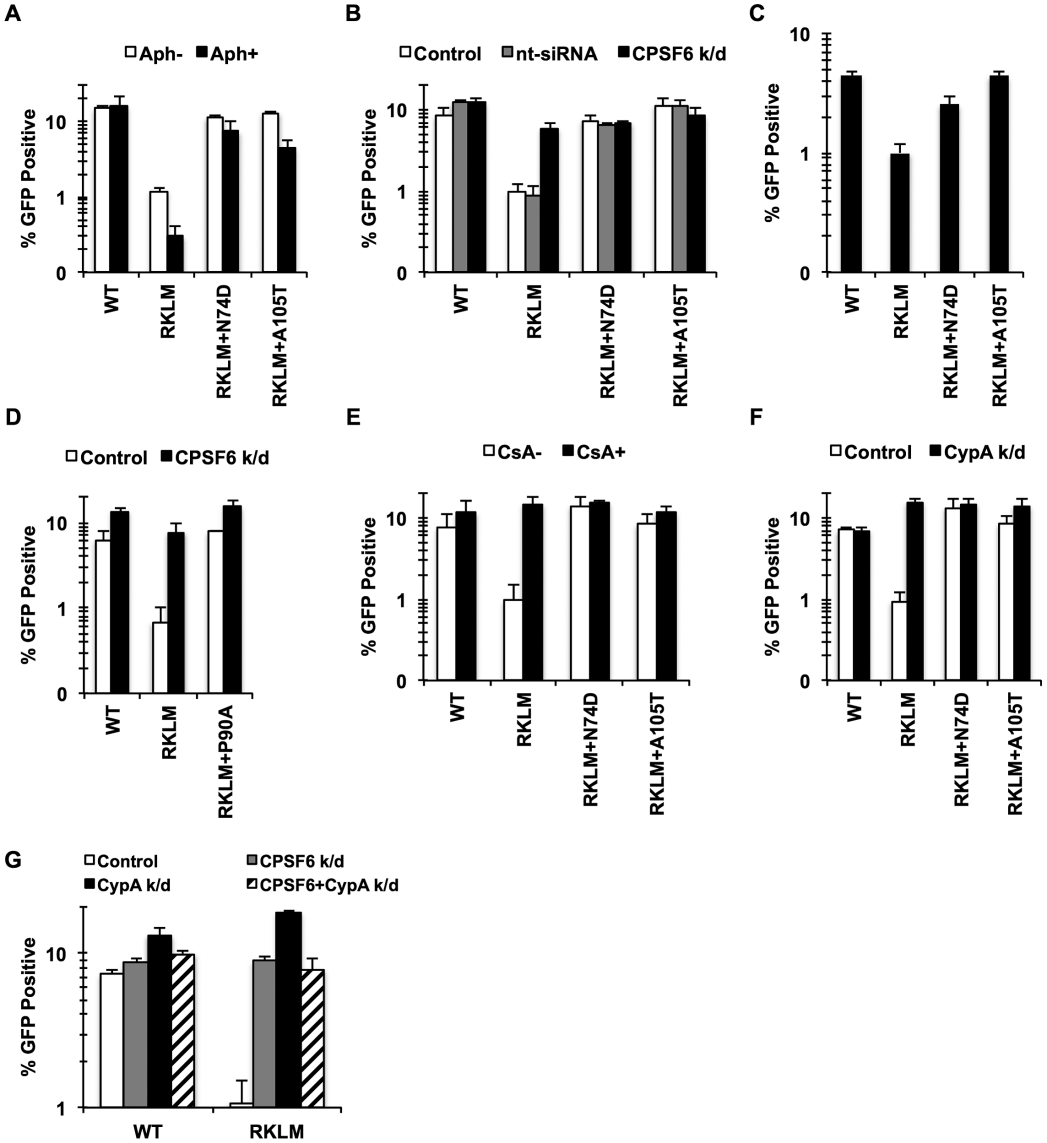
CPSF6 and CypA coordinate to suppress early HIV-1 CTL escape mutants in HLA-B27+ subjects. (A) HeLa cells were infected with RT-normalized VSV-G-pseudotyped GFP reporter viruses in the presence or absence of aphidicolin (Aph). Viral infectivity by RKLM and RKLM+A105T was significantly lower in Aph-treated cells than in non-treated cells (p<0.05). (B) VSV-G-pseudotyped GFP reporter viruses were used to infect HeLa cells after siRNA knockdown of CPSF6. CPSF6 depletion significantly increased infectivity of RKLM (p = 0.002). RKLM was different from both double mutants in responding to CPSF6 depletion (p<0.002). (C) Activated primary CD4+ T cells were infected with RT-normalized VSV-G-pseudotyped GFP reporter viruses. Increase in viral infectivity of both double mutants relative to RKLM was statistically significant (p<0.01). (D) VSV-G-pseudotyped GFP reporter viruses were used to infect HeLa cells after siRNA knockdown of CPSF6. (E) RT-normalized VSV-G-pseudotyped GFP reporter viruses were used to infect HeLa cells in the presence or absence of CsA. Relative to CsA-untreated cells, CsA significantly increased infectivity of RKLM (p<0.01). Response of RKLM to CsA treatment differed from that of double mutants (p<0.001). (F) RT-normalized VSV-G-pseudotyped GFP reporter viruses were used to infect HeLa cells after siRNA knockdown of CypA. Infectivity of RKLM was higher in CypA-depleted cells than in control cells (p<0.01). RKLM differed from double mutants in its sensitivity to CypA depletion (p<0.001). (G) HeLa cells transfected with siRNA against CypA, CPSF6 or both simultaneously were infected with RT-normalized VSV-G-pseudotyped GFP reporter viruses. Results are one representative experiment of at least two experiments, except for A where the average of viral infectivity from four independent experiments was plotted with the standard error of the mean. Standard deviations of a single triplicate experiment are indicated with the error bars.

To address the physiological relevance of the novel inhibitory function of CPSF6 for HIV-1 CA mutants, we extended our study to CTL escape mutations (R132K/L136M; hereafter called RKLM) in HLA-B27+ individuals. The R132K mutation occurring at the HLA-B27 anchor position −2 prevents the binding of B27 to the peptide and thus allows escape from HLA-B27-restricted CTL response [45]. We became interested in these CA mutations because Qi et al. [39] showed that infection by HIV-1 encoding the R132K substitution, which shares similar properties with RKLM [43], is also inhibited by aphidicolin treatment of cells (**Fig. 3A**). Because of its phenotypic similarity to T54A, we hypothesized that the RKLM virus is also restricted by CPSF6. In support of this, depletion of endogenous CPSF6 markedly enhanced the infectivity of the RKLM mutant to nearly WT levels (**Fig. 3B** **; p = 0.0002**). Moreover, addition of the N74D or A105T substitutions in the CPSF6-binding pocket increased the infectivity of RKLM in non-dividing cells (**Fig. 3A**). Upon infection with RT-normalized viruses, overall infectivity defects were restored even in dividing HeLa cells (**Fig. 3A** **; p<0.001**). The triple mutants RKLM+N74D and RKLM+A105T were no longer sensitive to CPSF6-mediated restriction, as they did not exhibit any increase in infectivity upon CPSF6 depletion (**Fig. 3B**). We next determined whether endogenous CPSF6 inhibits RKLM in primary CD4+ T cells as a more physiological relevant cell type. Infection by RKLM was reduced 3-fold compared to WT (**Fig. 3C** **; p = 0.03**). Addition of mutations in the CPSF6 binding pocket (N74D or A105T) almost completely rescued viral infectivity of RKLM in activated primary CD4+ T cells (**Fig. 3C** **; p<0.01**). Thus, the antiviral effect of CPSF6 is active against virus carrying naturally occurring mutations in relevant targets of HIV-1 infection *in vivo*.

Previous studies showed that CypA contributes to cell cycle-dependent infection by HIV-1 CA mutants [39]–[41], [46], including RKLM [39]. To determine whether CPSF6 and CypA block these viruses independently or together, we investigated their restrictive effects in a condition where one of the two molecules is prevented from interacting with viral capsids. Blocking CypA-CA interactions through a genetic mutation (P90A) (**Fig. 3D**) or by addition of CsA (data not shown) rendered RKLM less sensitive to the inhibitory function of CPSF6. As previously reported [43], infection by RKLM was enhanced by neutralizing the inhibitory action of CypA by blocking CypA-CA interactions (by either addition of CsA or CypA depletion) (**Fig. 3E, 3F**). Moreover, blocking CPSF6-CA interactions through genetic mutations (N74D and A105T) rendered RKLM unresponsive to the inhibitory action of CypA (**Fig. 3E, 3F**). Double knockdown of CypA and CPSF6 did not have an additive effect on the restoration of RKLM (**Fig. 3G**). Similar observations were obtained with the T54A CA mutant (**Fig. S4**), except that T54A was less responsive to CPSF6 depletion. These observations uncover the functional crosstalk between CypA and CPSF6 in thwarting infection of cell cycle-dependent CA mutants. As an initial approach to determine whether CypA promotes CPSF6 binding to the HIV-1 capsid, we performed an *in vitro* binding experiment in which CA-CypA interactions were prevented by addition of CsA. However, the presence or absence of CsA did not detectably alter the ability of CPSF6 to bind to CA tubes, suggesting that CypA and CPSF6 bind independently to the HIV-1 capsid (**Fig. S4E**).

**Figure 4:**
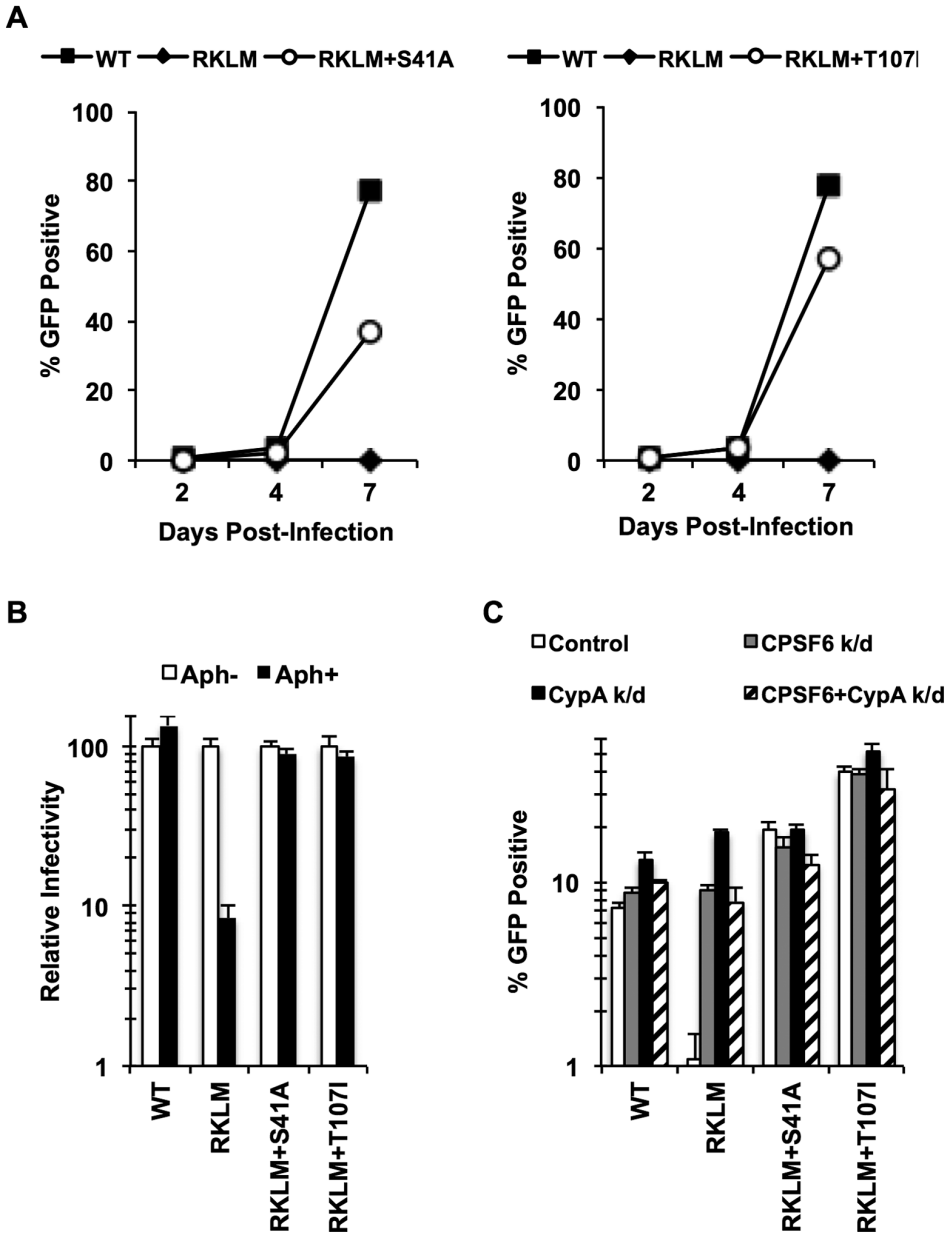
*In vitro* adaptation of the early HIV-1 CTL escape variant RKLM selects for a CPSF6-resistant virus. (A) CEM cells were infected with replication-competent GFP reporter viruses for spreading infections. (B) HeLa cells were infected with VSV-G-pseudotyped GFP reporter viruses in the presence or absence of aphidicolin (Aph). Both double mutants were different from RKLM in their ability to infect aphidicolin-treated cells (p<0.02). (C) HeLa cells transfected with siRNA against CPSF6, CypA or a combination of the two were infected with VSV-G-pseudotyped GFP reporter viruses. RKLM, but not the two double mutants, was significantly rescued by either CPSF6 or CypA depletion (p<0.01). Results are one representative experiment of at least three independent experiments, except for the double knockdown experiment in C. Standard deviations of a single triplicate experiment are indicated with the error bars.

**Figure 5:**
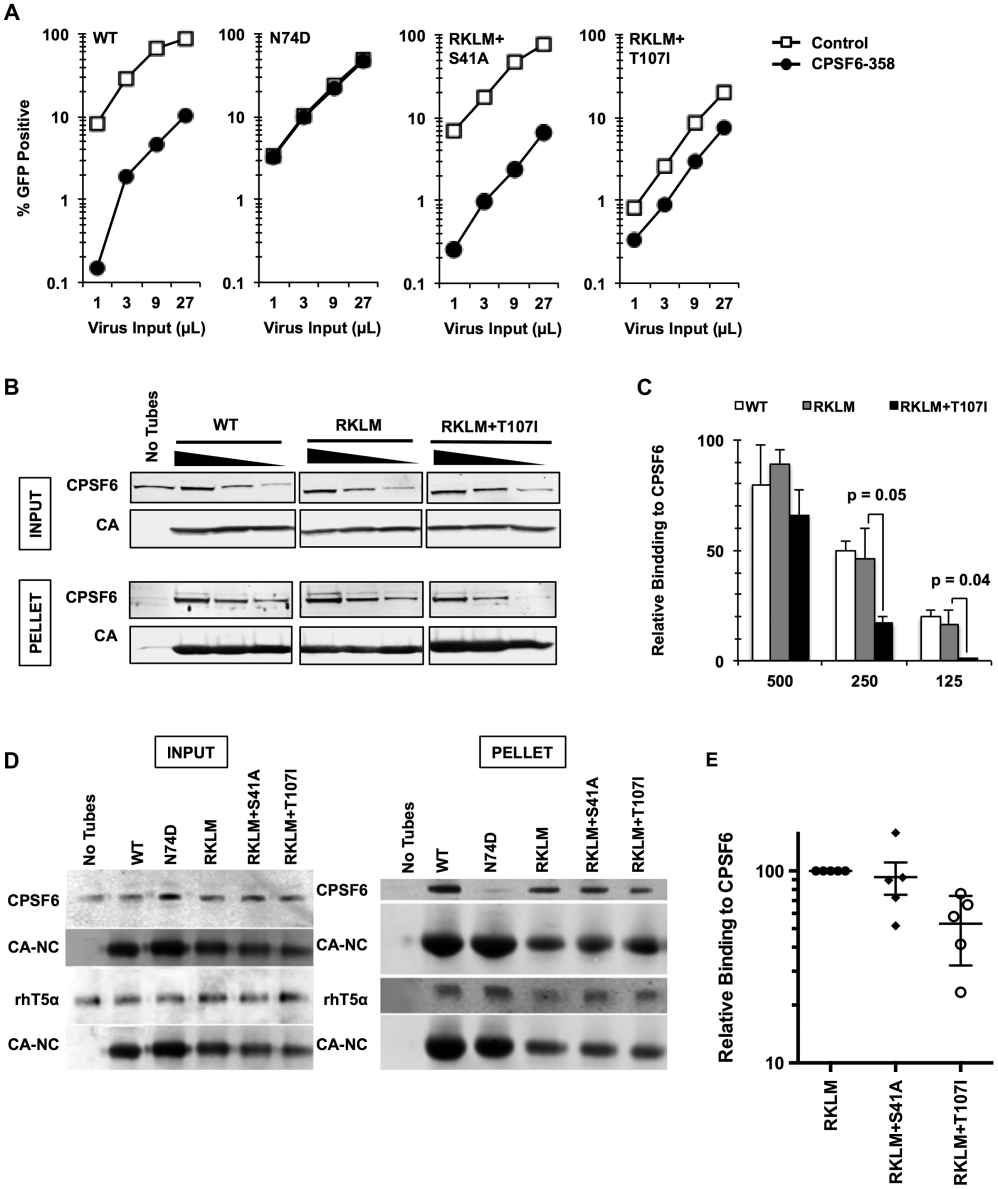
*In vitro*-derived CPSF6-resistant virus may alter CPSF6 binding. (A) HeLa cells stably transduced with the control vector LPCX or one overexpressing CPSF6-358 were infected with VSV-G-pseudotyped GFP reporter viruses. Results are one representative experiment of at least three independent experiments. (B) WT, RKLM and RKLM+T107I CA tubes were incubated with HeLa cell lysates as described in the Materials and Methods section. Complexes were pelleted and analyzed by quantitative immunoblotting for CPSF6 and CA. Input represents 10% of each reaction withdrawn prior to pelleting. The three individual panels shown for each protein are from one scan of a single immunoblot and were not manipulated. (C) Average of non-normalized data from three independent experiments that utilize HIV-1 CA tubes. One representative result is shown in B. Values represent the ratio of CPSF6 to CA in the pelleted CA complexes. The difference in CPSF6 binding between RKLM and RKLM+T107I was significant at lower concentrations of lysates. P values were determined by unpaired t-test. (D) HIV-1 CA-NC tubular complexes were incubated with cell lysates either from 293T cells transfected with HA-tagged rhesus TRIM5α (rhT5a) or HeLa cells for endogenous CPSF6. Ultracentrifugation through a 60% sucrose cushion was used to pellet HIV-1 CA-NC tubes and associated proteins. A small amount of the mixture was saved before ultracentrifugation and used as input. We have noted that HIV-1 CA-NC tubes carrying the RKLM mutations have reduced amounts of CA-NC in the pelleted fraction. At least two independent experiments were performed for CA binding to rhesus TRIM5α while five experiments were done for CA binding to CPSF6. One representative data for each experiment is shown here. (E) Summary of binding experiments using HIV-1 CA-NC tubes for three mutants. One representative result is shown in D. Binding of mutants carrying either RKLM+S41A or RKLM+T107T to endogenous CPSF6 is shown as relative to binding of CPSF6 to RKLM CA-NC. The level of CPSF6 in the pelleted fraction measured by LICOR was normalized by the level of CA-NC. The difference between RKLM and RKLM+S41A was not statistically significant (p = 0.71) whereas the difference between RKLM and RKLM+T107I was significant (p<0.01). The mean and standard error of the mean of five independent experiments are shown by horizontal bars.

To gain further insight into the mechanism of the CPSF6-mediated restriction of HIV-1 CA mutants, we adapted the RKLM mutant in immortalized CD4+ T cell lines. Serial passages of the RKLM selected two independent viruses that are highly improved in their replicative capacity. Sequence analysis of the CA region of these two adapted viruses did not reveal any reversion of RKLM to the WT sequence but instead one novel amino acid replacement in each of the two adapted viruses: S41A and T107I. While S41A is a common compensatory mutation for RKLM arising in HLA-B27+ individuals [43], T107I is of particular interest because it is located within the CPSF6 binding pocket [36]. Introduction of each mutation into the molecular infectious clone of RKLM restores viral replicative capacity of RKLM in CEM cells (**Fig. 4A**) as well as the ability to infect non-dividing cells (**Fig. 4B**). These two adaptive mutations (S41A or T107I) confer resistance to the CPSF6-mediated restriction, as the RKLM virus carrying either of these mutations was not rescued upon depletion of CPSF6 and CypA independently or simultaneously (**Fig. 4C**). T107 directly participates in binding with V314 of CPSF6 [36]. While neither mutation alone (S41A or T107I alone) alters HIV-1 sensitivity to CPSF6-358 restriction (**Fig. S5A**), we observed that RKLM+T107I is less sensitive to CPSF6-358 restriction than WT virus (**Fig. 5A** **and S5B; p<0.0001**). Importantly, the phenotype of RKLM+T107I resembles that of T107A; T107A was shown to have reduced affinity to the short peptide from CPSF6 and remained slightly sensitive to CPSF6-358 restriction [36]. These observations suggest that T107I reduces the binding of CA to CPSF6. In support of this hypothesis, both HIV-1 CA tubes and HIV-1 CA-NC tubes bearing RKLM+T107I substitutions in CA exhibited reduced binding (∼50%) to CPSF6 *in vitro* (**Fig. 5B, 5C, 5D, 5E**), while all of the HIV-1 CA-NC tubes had similar levels of binding to rhesus TRIM5α (**Fig. 5D**). This reduction in CA binding to CPSF6 also parallels the observation with T54A+A105T, which also exhibits reduced binding to CPSF6 (**Fig. 1B and 1C**). Therefore, these findings support the idea that inhibiting CA binding to CPSF6 is a common mechanism for cell cycle-dependent HIV-1 CA mutants to overcome CPSF6-mediated restriction. Taken together, we demonstrate that endogenous CPSF6 hampers infection of naturally occurring HIV-1 CA variants, possibly through binding to incoming viral capsids together with CypA.

### HIV-1 evades the inhibitory action of CPSF6 while preserving CA-CPSF6 interactions *in vivo*

The inhibitory function of CPSF6 was puzzling given the high degree of conservation of CPSF6 binding among diverse lentiviruses [36], [47]. As N74D, one of the CPSF6-independent viruses, is defective in primary macrophages [19], [48], CPSF6 may possess a beneficial function that is required specifically for HIV-1 replication *in vivo*. To further delineate the functional role of CPSF6 in HIV-1 replication, we exploited the unique pattern of CA evolution in HLA-B27+ subjects [43]. As described above, the RKLM mutations allow viral escape from the CTL response, yet the same RKLM mutation would render the virus sensitive to CPSF6-mediated restriction. The consequences of these two opposing virus-host interactions are an effective control of viremia in HLA-B27+ subjects. However, similar to our *in vitro* adaptation, *in vivo* evolution generates late CTL variants that can sustain high levels of viral replication in these individuals [43]. We asked whether and how these late variants escape from CPSF6-mediated restriction.

**Figure 6:**
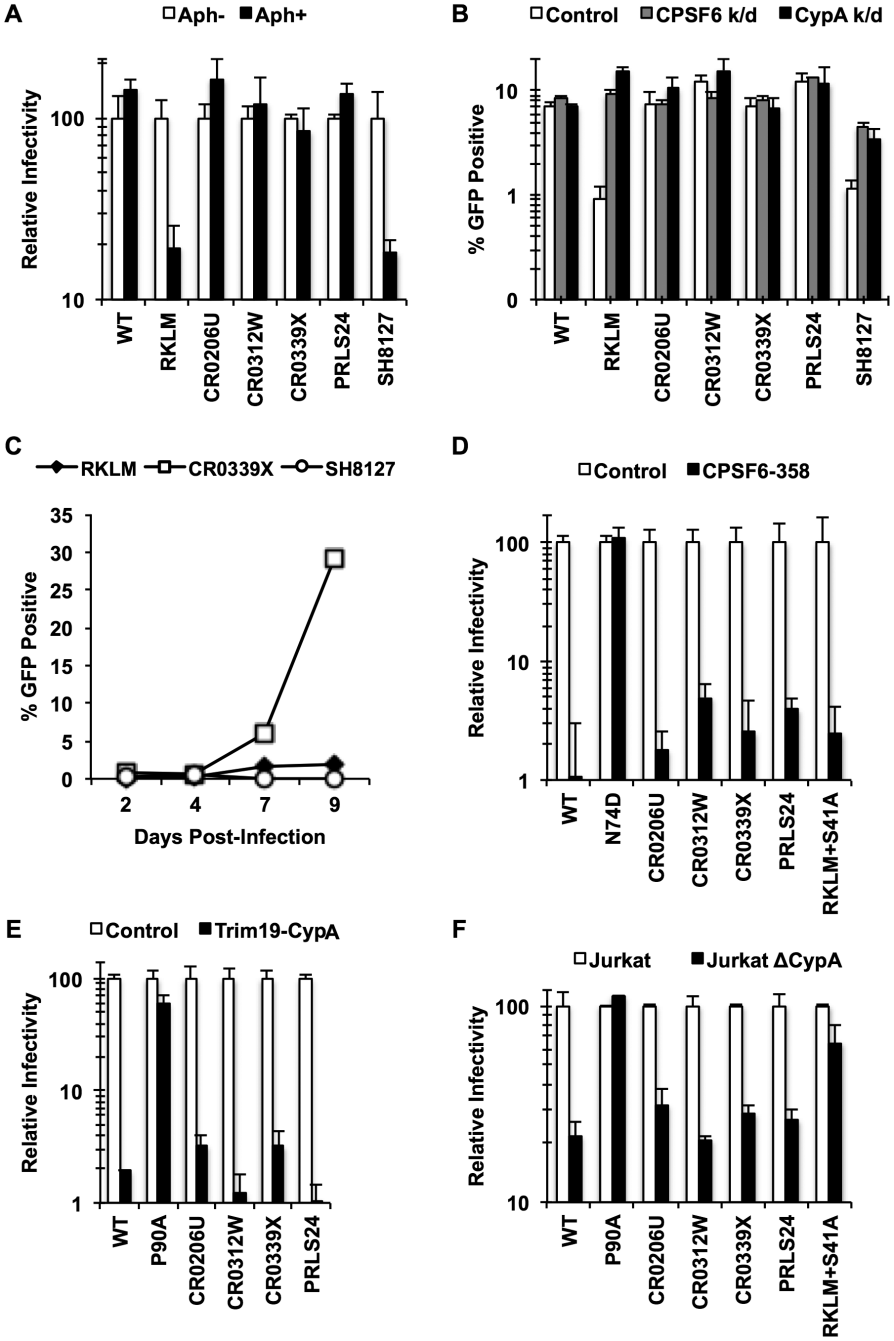
HIV-1 CTL escape variants invariably preserve CA-CPSF6 interactions *in vivo* during their evasion from the restrictive action of CPSF6. (A) HeLa cells were infected with VSV-G-pseudotyped GFP reporter viruses in the presence or absence of aphidicolin (Aph). Only RKLM and SH8127 were significantly blocked in aphidicolin-treated cells (p<0.05). (B) HeLa cells were transfected with siRNA targeting CPSF6 or CypA and infected with VSV-G-pseudotyped GFP reporter viruses. All the “*in vivo*” viruses were less sensitive to CPSF6 or CypA depletion (p<0.02). However, among these “*in vivo*” viruses only SH8127 had significant increase relative to control upon CPSF6 or CypA depletion (p<0.05). (C) CEM cells were infected with replication-competent GFP reporter viruses for spreading infections. (D) VSV-G-pseudotyped GFP reporter viruses were used to infect HeLa cells stably transduced with the control vector LPCX or one overexpressing CPSF6-358. (E) VSV-G-pseudotyped GFP reporter viruses were used to infect control HeLa cells or those stably overexpressing TRIM19-CypA. (F) Control Jurkat cells and those lacking CypA were infected with VSV-G-pseudotyped GFP reporter viruses. Results are one representative experiment of at least two triplicate experiments. Standard deviations (except for F) or standard errors of the mean of a single experiment are indicated with the error bars.

We generated chimeric viruses carrying the entire capsid sequence from five HLA-B27+ subjects infected with HIV-1 [49] (**Fig. S6**). All of these CA sequences contain the R132K substitution, which confers escape from CTL response but sensitivity to CypA-mediated restriction [39], [43], which is identical to CPSF6-mediated restriction. As described above, one notable property accompanying escape from CPSF6-mediated restriction is the restorated ability to infect non-dividing cells. We find that all of the chimeric viruses, except for SH8127, retained this ability, as opposed to the “early” CTL escape mutant just carrying RKLM (**Fig. 6A**). These same four viruses also appeared to acquire resistance to CPSF6-mediated restriction, as they were no longer enhanced by depletion of either CPSF6 or CypA, while like RKLM, SH8127 infectivity was enhanced by CPSF6 depletion (**Fig. 6B**). Finally, CR0339X, the only replication-competent virus we generated among the four adapted viruses, replicated efficiently in CEM cells, while SH8127 was defective (**Fig. 6C**). These data suggest that all capsid sequences, except for SH1827, adapted *in vivo* by escaping CPSF6-mediated restriction. Notably, CPSF6 escape correlates well with viral loads *in vivo*. All of the reported subjects had high viral loads (179,000–750,000 viral RNA copies per mL) [49]; this is in contrast to the subject from which SH8127 was isolated, who exhibited minimal detectable plasmid viral RNA. Previous reports demonstrated that mutations at S41 in the CA sequence restore *in vitro* replicative capacity to CTL escape variants carrying the signature RKLM mutation [43]. Consistent with this *in vitro* observation, mutations at S41 are highly associated with increased viral loads in HLA-B27+ individuals [43], [49]. While all four adapted CA sequences contain mutations at S41, SH8127 did not bear any genetic change at this position (**Fig. S6**). In fact, when S41 was restored in three of the four adapted CA sequences, two of them regained the sensitivity to restriction by CPSF6 (**Fig. S7**), underscoring the role of this position in sensitivity to CPSF6-mediated restriction. Taken together, albeit with the small sample size, the sensitivity to CPSF6-mediated restriction and viral loads in HLA-B27+ subjects is tightly correlated.

We next asked how these late variants escape from CPSF6-mediated restriction. Our *in vitro* adaptation experiments showed that RKLM can escape from this restriction by modulating the ability of CA to interact with CPSF6 (i.e. RKLM+T107I). However, none of the four adapted CA sequences selected for *in vivo* have any mutations at amino acids that participate directly in binding to the CPSF6 peptide [49] (**Fig. S6**). In fact, we find that chimeric viruses carrying these capsid sequences were effectively blocked by CPSF6-358 (**Fig. 6D** **; p<0.01**), suggesting that they still maintain CPSF6 binding. As described earlier (**Fig. 3E, 3H**), preventing CA binding to CypA rescues viruses sensitive to CPSF6-mediated restriction. Thus, such sensitive viruses could potentially escape from the CPSF6-mediated restriction by modulating the binding to CypA. However, these four adapted viruses, some of which contain mutations in CypA-binding loop [32], [50] (**Fig. S6**), also retain the binding to CypA, as evident by their sensitivity to the fusion protein between TRIM19 and CypA (TRIM19-CypA) (**Fig. 6E** **; p<0.01**). While this genetic assay with TRIM19-CypA is convenient to detect the binding of CA to CypA, it may not fully recapitulate physiologically relevant CA-CypA interactions that promote HIV-1 infection. To circumvent this potential issue, functional interaction with CypA was confirmed with the CypA-null Jurkat cell line in which CypA-dependent viruses were all blocked relative to the control Jurkat cell line (**Fig. 6F** **; p<0.05**). As described above, all four adapted viruses contain compensatory mutations at S41 that are almost invariably found among other late variants in HLA-B27+ subjects. The RKLM mutant carrying S41A, which was incidentally isolated in our *in vitro* adaptation, also retained binding to CPSF6 (**Fig. 5A** **, Fig. S5B,** **Fig. 5D** **,** **Fig. 5E**). We noticed a trend in which RKLM+S41A binds to CPSF6 ∼7% weaker than RKLM; however, this was not supported by statistical significance (**Fig. 5E**). Interestingly, the same mutant (RKLM+S41A) was less dependent on CypA that WT for its infection (**Fig. 6F**). Altogether, these results reveal two opposing CA-dependent functions of CPSF6 during HIV-1 replication *in vivo*. CPSF6 exerts deleterious effects on the early CTL escape variants in HLA-B27+ subjects. Such a restricted virus has the potential to escape from CPSF6 by modulating CPSF6 binding (**Fig. 5**). However, *in vivo* evolution selected for viruses that escape from CPSF6-mediated restriction yet retain CPSF6 binding. This observation supports the idea that CPSF6 is beneficial for HIV-1 replication *in vivo*.

### Selective pressure for CPSF6 binding in primary CD4+ T cells mimics *in vivo* adaptation

**Figure 7:**
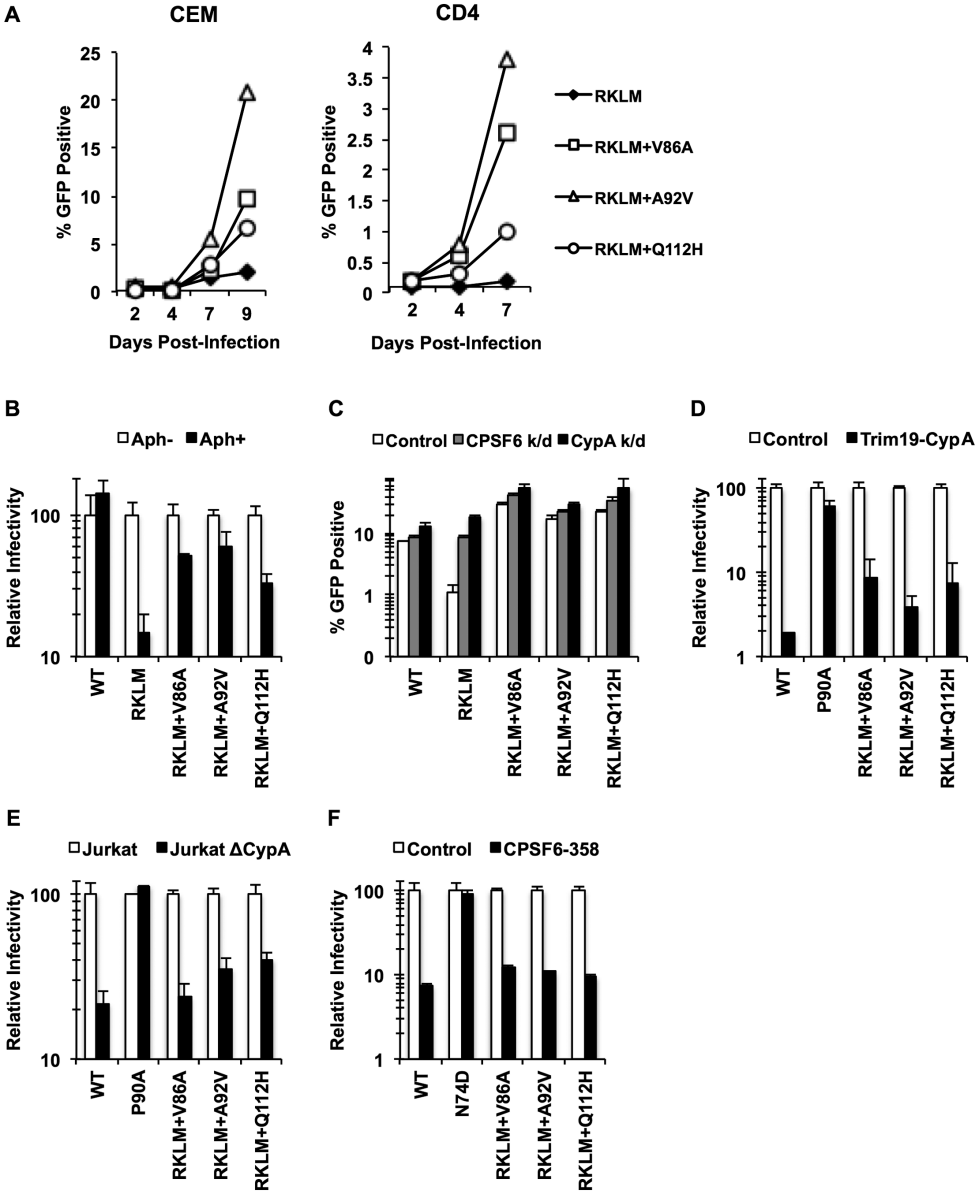
Adaptation of the early HIV-1 CTL escape variant RKLM in primary CD4+ T cells mimics *in vivo* adaptation. (A) Replication-competent GFP reporter viruses were used to infect CEM cells or primary activated CD4+ T cells. (B) HeLa cells were infected with VSV-G-pseudotyped GFP reporter viruses in the presence or absence of aphidicolin (Aph). RKLM+A92V and RKLM+Q112H were blocked in non-dividing cells with statistical significance (p<0.05) however they also differ from RKLM in their infectivity in aphidicolin-treated cells (p<0.05). (C) HeLa cells were transfected with siRNA against CPSF6 or CypA and then infected with VSV-G-pseudotyped GFP reporter viruses. The three “*ex vivo*-adapted” viruses were not as sensitive as RKLM in their sensitivity to CPSF6 or CypA knockdown (p<0.02). (D) VSV-G-pseudotyped GFP reporter viruses were used to infect control HeLa cells or those stably overexpressing TRIM19-CypA. The three adapted viruses were restricted in CPSF6-358-expressing cels (p<0.001). (E) Control Jurkat cells and those lacking CypA were infected with VSV-G-pseudotyped GFP reporter viruses. The three adapted viruses were blocked in Jurkat cells lacking CypA (p<0.01). (F) VSV-G-pseudotyped GFP reporter viruses were used to infect HeLa cells stably transduced with the control vector LPCX or LPCX encoding CPSF6-358. CPSF6-358 blocked all the adapted viruses (p<0.02). Results are one representative experiment of at least two experiments. Standard deviations (except for E) or standard errors of the mean of a single triplicate experiment are indicated with the error bars.

CPSF6 binding is strictly maintained *in vivo* but is dispensable for HIV-1 replication *in vitro*. Given that activated CD4+ T cells are the major cell type to produce HIV-1 *in vivo*, we investigated the requirement of CPSF6 for HIV-1 replication in primary CD4+ T cells by studying how the RKLM mutant will evolve in primary CD4+ T cells. Three independent experiments for experimental adaptation of the RKLM virus in this cell type generated three genetic changes (V86A, A92V and Q112H) in the viral capsid sequences without reversion of the parental RKLM mutations. We examined the sequence of only the CA region of these adapted viruses, but each of these three mutations, when separately introduced into the parental RKLM mutant, restored infectivity to RKLM in both CEM cells and primary CD4+ T cells (**Fig. 7A**). These three mutations confer resistance against CPSF6-mediated restriction, as the viruses containing each of the mutations regained the ability to infect non-dividing cells (**Fig. 7B**). Furthermore, they were not significantly rescued by CPSF6 or CypA knockdown, relative to RKLM (**Fig. 7C**). Two of these mutations, V86A and A92V, are located within the CypA binding loop [32]. In fact, RKLM carrying each of these three mutants was less sensitive to restriction by TRIM19-CypA than WT (**Fig. 7D** **, p<0.05**). However, their infectivity was impaired in CypA-null Jurkat cells similar to that by WT (**Fig. 7E**). More importantly, we find that they retain the ability to interact with CPSF6 by the CPSF6-358 restriction assay (**Fig. 7F**). Thus, *ex vivo* escape from CPSF6-mediated restriction takes a similar course as *in vivo* escape. In both settings, HIV-1 strictly maintains CPSF6 binding as opposed to *in vitro* adaptation.

### *In vivo* selection for CPSF6 binding by HIV-1 is linked to invariable utilization of TNPO3, RanBP2 and Nup153

**Figure 8:**
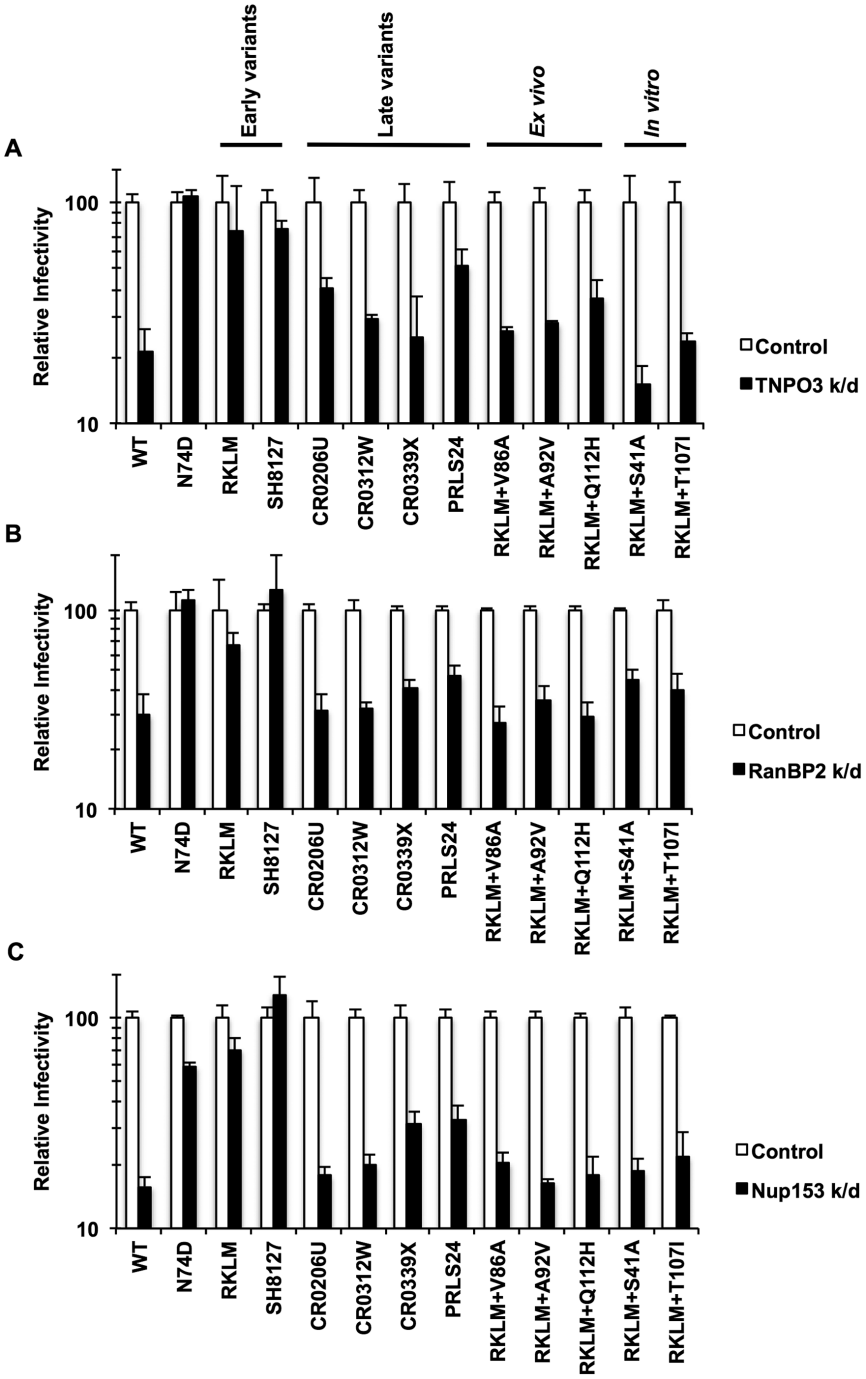
Dependence of various HIV-1 mutants on TNPO3, RanBP2 and Nup153. (A) HeLa cells were transfected with siRNA against TNPO3 and infected with VSV-G-pseudotyped GFP reporter viruses. (B) To deplete RanBP2, HeLa cells were first infected with a VSV-G-pseudotyped virus carrying a Crimson reporter and shRNA targeting RanBP2. They were then challenged with VSV-G-pseudotyped GFP reporter viruses. Viral infectivity shown in this data was determined by measuring GFP+ cells in crimson-positive populations. (C) HeLa cells were transfected with siRNA against Nup153 and infected with VSV-G-pseudotyped GFP reporter viruses. Results are one representative experiment of at least two experiments. Standard errors of a single triplicate experiment are indicated with the error bars.

What drives recurrent selection of CPSF6 binding *in vivo*? Price *et al.* proposed a new role for CPSF6 in the utilization of host factors implicated in HIV-1 nuclear entry, such as TNPO3, RanBP2 and Nup153 [36]. To explore this idea, we examined pre-adapted and adapted viruses for their dependence on these host molecules by depleting these gene products by RNAi. We find that the “early variants” (RKLM or the pre-adapted CTL escape mutant SH8127) were less sensitive or completely insensitive to depletion of these molecules (**Fig. 8**; p<0.05 when compared with WT), indicating that CPSF6-mediated restriction eliminates the ability to utilize these molecules. In contrast, all the adapted viruses *in vivo* (“late variants”) were blocked at levels comparable to the WT virus (**Fig. 8** **; p<0.05**). Therefore, escape from CPSF6-mediated restriction *in vivo* maintains CPSF6 binding and restores the ability to utilize these host molecules (**Fig. 8**). Again, all three *ex vivo*-adapted viruses mirror the *in vivo*-adapted viruses, since they were all dependent upon these nuclear entry factors (**Fig. 8** **; p<0.05**). Interestingly, RKLM+T107I, which is less sensitive to CPSF6-358 and resistant to CPSF6-mediated restriction, also regains the ability to utilize these nuclear entry factors (**Fig. 8** **; p<0.05**). In summary, these findings indicate that *in vivo* escape from CPSF6-mediated restriction that retains CPSF6 binding is strongly correlated with recovery of utilization of TNPO3, RanBP2 and Nup153.

## Discussion

Here we provide evidence for two divergent functions of CPSF6. We demonstrate that endogenous CPSF6 exerts a direct inhibitory effect on naturally occurring HIV-1 variants in individuals with HLA-B27. However, we find a strong selective pressure in these individuals to preserve CPSF6 binding while escaping from the restrictive activity by CPSF6, arguing for a beneficial role of CPSF6 for *in vivo* HIV-1 replication.

### Endogenous CPSF6 blocks cell cycle-dependent HIV-1 CA mutants

There is mounting evidence to suggest that CPSF6 plays a critical role in HIV-1 replication [18], [35], [36]; however, its exact function remains to be defined. In this study, we showed that depletion of endogenous CPSF6 rescues infection by HIV-1 CA mutants that are impaired for infection of non-dividing cells (**Fig. 2** **,** **3**). We also found that reducing CPSF6 binding by CA mutations in the CPSF6-binding pocket restores infectivity to HIV-1 CA mutants that are sensitive to CPSF6-mediated restriction (**Fig. 1** **,** **5**), suggesting that CPSF6-mediated restriction depends on direct binding of endogenous CPSF6 to incoming viral capsids. Previous studies, which employed *in vitro* binding assays, restriction by CPSF6-358, and cytoplasmic localization of CPSF6 by TNPO3 knockdown [18], [35], [36], indicate that CPSF6 can directly bind to the viral capsid. Our current data complement these observations and support the idea of the direct interaction between endogenous CPSF6 and incoming capsids in a physiological setting.

We observed that infection by CA mutants restricted by CPSF6 is less dependent or independent of the nucleoporins RanBP2 and Nup153. As these host proteins are components of the nuclear pore complex, we suggest that CPSF6 engages the viral capsid in the cytoplasm. In this respect, CPSF6 appears to resemble cyclophilin A (CypA), as CypA also binds to incoming viral capsids in the cytoplasm [32], [33]. Our data also reveal the functional crosstalk between CPSF6 and CypA. Specifically, CA mutants sensitive to CPSF6-mediated restriction are also inhibited by CypA [39], [41]–[44], [46], [51], [52]. Expression of CPSF6 is essential for CypA-mediated restriction, which parallels CPSF6-mediated restriction (**Fig. 3** **, Fig. S4**). Mutations in CA that result in cell cycle-dependent infection may alter the normal course of viral uncoating. In this model, CypA and CPSF6 may exacerbate such an uncoating defect exhibited by cell cycle-dependent CA mutants. This is consistent with a recent finding that reveals core stabilization as a mechanism of restriction of the WT virus by cytoplasmic CPSF6 [35], [37]. CypA has also been reported to control HIV-1 uncoating both *in vitro* and in target cells [34], [46]. Other studies suggest a potential direct role for TNPO3 in HIV-1 uncoating [27], [34], thus it may be possible that TNPO3 is also involved in the inhibitory action of CPSF6 and CypA for cell cycle-dependent HIV-1 CA mutants, although this hypothesis appears inconsistent with our observation that TNPO3 depletion did not enhance infectivity of RKLM (**Fig. 8**).

### CPSF6 suppresses CTL escape mutants in HLA-B27+ subjects

The antiviral action of CPSF6 is physiologically relevant to HIV-1 replication *in vivo*, because we find that CPSF6 inhibits infection of early CTL escape variants in HLA-B27+ individuals (**Fig. 3** **,** **6**). Moreover, our data reveal a positive correlation between sensitivity to CPSF6-mediated restriction and effective suppression of viral replication *in vivo*. Namely, one capsid (SH8127) susceptible to CPSF6 restriction originated from a subject with very low viral loads (50 viral RNA copies per mL) [49], whereas those resistant to CPSF6 restriction were derived from individuals harboring much higher viral loads (179,000–750,000 viral RNA copies per mL) [49]. Furthermore, compensatory mutations at S41 that confer resistance to CPSF6-mediated restriction are significantly associated with higher viral loads in HLA-B27+ individuals [43], suggesting that escape from CPSF6-mediated restriction is essential for robust HIV-1 replication in these individuals. Naturally, this parallels the observation made by Schneidewind et al. who found that CypA is inhibitory towards early CTL escape variants [43]. Interestingly, mutations associated with HLA-B27 also increase sensitivity of HIV-1 to human TRIM5α [53]. Hence, inhibitory functions of these CA-binding host proteins contribute to the constraints that render CA immunologically vulnerable [54].

### HIV-1 actively preserves CPSF6 binding *in vivo* while escaping from restriction by CPSF6

The inhibitory function of CPSF6 was somewhat unexpected given the conservation of the CPSF6-binding pocket in CA proteins from diverse primate lentiviruses [36], [47]. However, the current study also provides evidence for an opposing, beneficial role of CPSF6 for HIV-1 replication *in vivo*. This was possible because of the unique pattern of HIV-1 CA evolution in HLA-B27+ subjects. Our data reveal the strong selective pressure for maintaining CPSF6 binding to CA in these subjects (**Fig. 6**). This is significant because this strict preservation of CPSF6 binding *in vivo* occurs despite the opposing pressure to reduce CPSF6 binding in order to escape from its inhibitory activity, as shown *in vitro* (**Fig. 5**). As described above, HIV-1 evolution in HLA-B27+ individuals takes one major escape pathway utilizing substitutions at S41 to evade CPSF6-mediated restriction. Thus, despite the small sample number in this study, it is likely that the preservation of CPSF6 binding is an inevitable requirement for adaptation of HIV-1 in these individuals. Therefore, these findings suggest that CPSF6 has an alternative role that is advantageous to HIV-1 replication *in vivo*.

### *In vivo* HIV-1 evolution restores utilization of TNPO3, RanBP2 and Nup153

What is the driving force to select for CA that binds CPSF6 *in vivo*? One proposed function of CPSF6 during HIV-1 infection is that CPSF6 controls the nuclear entry pathway for HIV-1 [36]. Consistent with this model, the strict preservation of CPSF6 binding *in vivo* was highly correlated with utilization of TNPO3, RanBP2 and Nup153 (**Fig. 8**). Namely, we observed that early CTL escape variants that lack adapted mutations at S41 are less dependent on TNPO3 and completely independent from RanBP2 and Nup153, but all of the late adapted viruses regained the ability to utilize these three cellular molecules. These observations suggest that utilizing TNPO3, RanBP2 and Nup153 is a major selective pressure for the maintenance of CA binding to CPSF6 *in vivo*. We propose that CPSF6 interactions with cell cycle-dependent mutants prevent the viral core from accessing the cellular nuclear entry machinery, and that mutations reducing CPSF6 binding relieve the impairment. As CPSF6 also binds the wild type HIV-1 capsid but does not inhibit infection by the wild type virus, it is possible that the RKLM substitutions weaken the interaction of the viral capsid with the nuclear pore, rendering the capsid sensitive to masking by CPSF6. Interestingly, while T107I and N74D substitutions in CA appear to rescue RKLM by reducing the binding to CPSF6, the S41A substitution rescued RKLM from CPSF6 inhibition without significantly altering the extent of CPSF6 binding (**Fig. 5**). Thus, S41A may relieve an uncoating defect induced by CPSF6 binding to the RKLM capsid, or it may enhance subsequent interactions with the nuclear pore proteins thereby circumventing the CPSF6 block. Studies of the interaction of the RKLM capsid with nucleoporins should help address this issue.

### Cell type-specific requirement of CPSF6 for HIV-1 replication

CPSF6 binding by HIV-1 is strictly conserved *in vivo* but flexible *in vitro*. For instance, *in vitro* escape of cell cycle-dependent CA mutants from the antiviral function of CPSF6 results in mutations (A105T and T107I) in the CPSF6 binding pocket [44] (**Fig. 4**), which reduce the CA-CPSF6 interactions (**Fig. 1** **,** **5**) [35]. Similarly, HIV-1 evasion from an antiviral form of CPSF6 selected for the first CPSF6-binding deficient mutation (N74D) [18]. The difference between *in vitro* and *in vivo* in their ability to accommodate genetic variability within the CPSF6-binding pocket is somewhat similar to the recent observation that some CA mutations with high *in vitro* fitness did not occur in natural HIV-1 subtype B populations [55]. However, the virus carrying the N74D mutation is severely attenuated in macrophages [19], [48], pointing to the possibility that the dependence of HIV-1 on CPSF6 may be cell type-specific. These observations may be clinically relevant. PF-3450074 is a recently described anti-HIV-1 compound that binds to the CPSF6-binding domain of the capsid. HIV-1 was shown to gain resistance against this compound *in vitro* by allowing mutations within the same binding domain [56], [57], but the escape barrier for such drugs would likely be high in HIV-1-infected individuals, since the CPSF6-binding domain of CA is immutable *in vivo*.

In this study, we demonstrated that the host protein CPSF6 possesses two opposing functions for HIV-1 replication *in vivo*. Viral evolution in HLA-B27+ subjects suggests a beneficial function of CPSF6 for HIV-1 replication *in vivo* that outweighs its inherently deleterious potential. This unique CPSF6-HIV interplay illustrates the complex nature of host-pathogen interactions. Physical contact of viral molecules with cellular components is one fundamental feature for viral hijacking of the host machinery, while at the same time host factors may become detrimental to viral replication. In this respect, the CPSF6-CA interaction may not be completely unique as a recent comprehensive study revealed additional HIV-human protein interactions that can attenuate HIV-1 replication [58].

## Materials and Methods

### Cloning

Molecular infectious clones based on the LAI strain were generated by introducing mutations into either a replication-competent pBru3oriGFP3 backbone [11], one that is Env-defective, or both using standard cloning procedures. Entire sequences of the capsid-encoding segment of HIV-1 Gag from five different infected individuals carrying the HLA-B27 haplotype, which were previously reported by Wang et al. [49], were synthesized (Life Technologies) and cloned into the above-mentioned molecular infectious clones. The E2-Crimson gene was cloned into the *nef* position of the wild-type *env*-deficient HIV-1 clone to generate pBru3ori-ΔEnv-Crimson. An HIV-1 Gag-Pol expression vector, pCRV1-Gag-Pol (LAI), was a derivative of pCRV1-Gag-Pol (gift from the Hatziioannou and Bieniasz labs) constructed by replacing the most of the Gag encoding sequence with that from LAI (from the Gag start codon to the unique ApaI site in LAI). Various CA mutations were introduced into this derivative by using standard cloning techniques. The gene depletion vector pLKO.1-Crimson-RanBP2 that contains shRNA targeting RanBP2 [19] based on the previous target sequence was generated by replacing the puromycin resistance gene of the parental pLKO.1 vector with the E2-Crimson gene amplified from the pTEC19 plasmid (Addgene). An HIV-1 CA-NC expression vector, pWISP96-18 (a kind gift of Wesley Sundquist) [59], was used as a template to introduce different HIV-1 CA mutations by overlapping PCR.

### Cell culture

HeLa cells were cultured in DMEM (Cellgro) and supplemented with 10% FBS (Cellgro) and 1× penicillin-streptomycin (P/S, Cellgro). Immortalized suspension cells (MT4, CEM, Jurkat) were cultured in RPMI (Cellgro) and supplemented with 10% FBS (Sigma), 1× P/S and 1× 2-glutamine (Cellgro). PBMCs were isolated from whole blood obtained from anonymous blood donors (New York Blood Center) using standard Ficoll (Cellgro) procedures. Primary CD4+ T cells were isolated using the Human CD4+ T Cell Enrichment Kit per manufacturer instructions (Easysep). Primary CD4+ T cells were activated using Dynabeads Human T-Activator CD3/CD28 (Gibco) and cultured in suspension cell medium supplemented with 30 units per mL of IL-2 (Peprotech). JurkatΔCypA cells [60] and HeLa cells overexpressing either TRIM19-CypA cells [40] or CPSF6-358 (gift by V. KewalRamani) [18] were previously described.

### siRNA knockdowns

HeLa cells were plated at 5×10^5^ cells per well of a 6-well plate and transfected with 30 pmol siRNA using Lipofectamine RNAiMAX (Invitrogen, 13778) or with the transfection reagent alone (control). Non-transfected cells were used as controls in all experiments. Non-targeting siRNA were also used in certain experiments as additional controls. We found no significant difference in viral infectivity between these controls. Specific siRNA used for knockdowns were: anti-CPSF6 (Thermo Scientific J-012334-11, J-012334-09), anti-CypA (GAUGAACUUCAUCCAGACUUU), anti-TNPO3 (Thermo Scientific L-019949-01-0010) and anti-Nup153 (GGACUUGUUAGAUCUAGUUUU). The following day the transfection was repeated with the same procedure. Four hours post-transfection, cells were seeded at 5×10^5^ cells per 96-well plate for infection. For RanBP2 knockdown experiments, HeLa cells plated at 5×10^5^ cells per well of a 6-well plate were infected with VSV-G-pseudotyped Crimson reporter viruses carrying RanBP2-targeting shRNA. Effects of gene depletion were analyzed by western blotting (**Fig. S8**). Two days after infection, 5×10^5^ cells were plated onto 96-well plates for a second infection with GFP reporter viruses.

### Infections

All viruses were generated using 293T cells as previously described [11] using polyethylenimine (PEI, PolySciences) as the transfection reagent. pBru3oriGFP3ΔEnv viruses were pseudotyped with the VSV-G envelope. Viruses were normalized using the Lenti RT Activity Kit (Cavidi). All infections using HeLa cells were performed at 5×10^5^ cells per plate. HeLa cells treated with 2 µg per ml of aphidicolin (Aph, Sigma) were plated at 1.5×10^6^ cells per plate. Cyclosporine A (CsA, Sigma) was added to the culture during infection at 2 µM. Immortalized T Cell lines and primary CD4+ T cells were plated at 2.5×10^5^ cells per mL and 1×10^6^ cells per mL, respectively. Virus infectivity was examined by measuring crimson- and/or GFP-positive cells using a BD LSRII Flow Cytometer. Abrogation experiments were done by co-infecting CPSF6-358-expressing HeLa cells with a fixed amount of HIV-1 WT virus (encoding the E2 crimson) and increasing amounts of abrogating viruses carrying various CA mutations. We used the amount of Crimson reporter virus such that infection with a restricted WT virus generates 0.2–1% Crimson-positive cells without restriction-abrogating viruses. Abrogating particles were generated by transfecting three plasmid DNA (packageable GFP-encoding HIV-1 vector, HIV-1 Gag-Pol expression vector and VSV-G-encoding vector) [61]. Two days after infection, crimson-positive cells were measured as described above.

### Virus adaptation

MT4 cells (1.25×10^5^ per well of a 24-well plate) were infected with replication-competent virus carrying the RKLM mutation (3 ng of reverse transcriptase). Supernatant harvested at 16 days post-infection (dpi) was used to infect fresh MT4 cells. DNA was extracted from acutely infected MT4 cells at 1 dpi using the DNeasy Blood & Tissue Kit (Qiagen). PCR was performed to amplify the segment of the Gag gene encoding the CA protein using primers (HIV-1-LAI-CA-Fwd 5′-GCACAGCAAGCAGCAGCTGACACAGG; HIV-1-LAI-CA-Rev 5′-GCCTCTTTGCATCATTATGGTAGC). The amplified fragments were subjected to direct sequencing with the same PCR primers (Genewiz). The MT4-adapted CA sequence had a nucleotide switch at CA C310T (based on the LAI strain: K02013), which leads to a threonine-to-isoleucine change at residue 107 of the viral CA protein (T107I). CEM cells were infected at 1.25×10^5^ cells per well of a 24-well plate with the RKLM virus (13 ng RT) in the presence of 2 µM CsA, which was added to augment the initial infection. The adapted virus was harvested at 21 dpi and analyzed by sequencing of the CA-encoding segment. The CEM-adapted sequence had a T-to-G nucleotide change at 121 in the viral capsid sequence, resulting in to a serine-to-alanine change at 41 of the viral CA protein. All primary CD4+ T cell adaptation experiments were carried out using isolated CD4+ T cells activated with CD3/CD28 beads. Each independent experiment was performed using the same donor, with a total of 3 separate donors. CD4+ T cells were infected at 5×10^5^ cells per well of a 24-well plate with the RKLM virus (30 ng RT). At 3 dpi, infected CD4+ T cells were mixed one-to-one with freshly isolated and activated CD4+ T cells from the same donor. This process was repeated until the proportion of virus-infected cells, as judged by GFP positivity, reached 15%. Cell-free supernatants were then harvested, passaged further, collected once GFP positivity reached 15% and used to infect fresh MT4 cells. 1 dpi, DNA was extracted and analyzed by sequencing of the CA-encoding segment. In each of the three experiments, we found the following substitutions: V86A was a nucleotide switch at CA T257C, A92V was a nucleotide switch at CA C275T and Q112H was a nucleotide switch at CA G336T.

### Western blotting

Protein levels were determined using standard laboratory western blot protocols. The primary antibodies used were: anti-CPSF6 (Proteintech 15489-1-AP), anti-CypA (Thermo Scientific PA1-025), anti-TNPO3 (Abcam ab54353), anti-RanBP2 (Abcam ab2938), anti-tubulin (Sigma-Aldrich T6074). Protein levels of Nup153 were determined using anti-nuclear pore complex proteins antibody (a gift from the Blobel laboratory). The secondary HRP-conjugated antibodies used were anti-rabbit (Invitrogen 65-6120) and anti-mouse (Santa Cruz sc-2005). Western blotting of capsid-binding assays was performed by using fluorescent secondary antibodies and signals were quantitated with a LI-COR Odyssey scanner.

### Capsid-binding assay

#### i) Capsid-Binding assay with HIV-1 CA-NC tubes

Capsid-binding assay utilizing the HIV-1 CA-NC protein was carried out in a similar manner as described [62] with minor modifications. Briefly, recombinant HIV-1 CA-NC protein purified from *Escherichia coli* (*E. coli*) was used to form high-molecular-weight complexes by incubating 150 µM CA-NC protein with 30 µM (TG)25 DNA oligonucleotide in a solution of 50 mM Tris-HCl (pH 8.0) and 500 mM NaCl overnight at 4°C. 293T cell lines seeded at 3×10^6^ cells one day before transfection were transfected with 6 µg of the expression vectors pLPCX-rhTRIM5α-HA (Gift of J. Sodroski). Two days after transfection, the cells were detached with PBS containing 5 mM EDTA and resuspended in 1.2 ml of hypotonic lysis buffer (10 mM Tris-HCl [pH 8.0], 10 mM KCl, 1 mM EDTA, 0.5 mM DTT. The cells were placed on ice for 15 min and lysed by using a 1 ml Dounce homogenizer (Pestle B, 15 strokes). The cell debris was cleared by centrifugation at 4°C for 10 min at 14,000× *g*. Cell lysates were prepared from HeLa cells by the same procedure, except that the cells were sonicated four times for 15 seconds with 15 second pauses on ice instead of Dounce homogenization. One hundred twenty-five micrograms of the lysates were used for the binding assay while 5 µl of the assembled CA-NC complexes were mixed with the lysate with the concentration of NaCl adjusted to 150 mM. The reaction mixture was incubated for one hour at room temperature with gentile mixing every 10 min. After incubation, 10 µl of the sample was saved as input and the rest was layered onto a 3.5 ml 60% sucrose cushion (1× PBS, 0.5 mM DTT). The mixture was then centrifuged at 100,000× *g* in an SW50 rotor (Beckman) for 1 h at 4°C. After centrifugation, the sucrose cushions were carefully removed by aspiration and the pellet was dissolved in 50 µl of 1× SDS-loading buffer. Rhesus TRIM5α was detected by Western blotting with an anti-HA antibody (HA. 11 Clone 16B12, Covance). The level of CA-NC protein was examined with an anti-p24 CA antibody.

#### ii) Capsid-binding assay with HIV-1 CA tubes

Recombinant CA tubes were prepared from proteins expressed in *E.coli* as follows. Mutations were introduced into pet21a vectors encoding cysteine-mutated HIV-1 CA at positions 14 and 45 and purified from *E. coli* according to Pornillos *et al.* [63]. Purified proteins were then assembled by dialysis into Buffer 1 (1 M NaCl, 50 mM Tris pH 8, 20 mM 2-mercaptoethanol) for three hours, followed by dialysis into Buffer 2 (1 M NaCl, 50 mM Tris pH 8) overnight. CA assemblies (5 µM) were then incubated with HeLa cell extracts for one hour with gentle mixing in 100 µl reactions in binding buffer (50 mM Tris pH 8, 150 mM NaCl, 5 mM MgCl2, and 0.5 mM EDTA). When indicated, cyclosporine A (Calbiochem) was included at 5 µM concentration. Reactions were then centrifuged for five minutes at 5,000× *g* and the pellets washed with 100 µl of binding buffer before pelleting again. The pellets were analyzed by SDS-PAGE and immunoblotting with antibodies against CA (183-H12-5C), cyclophilin A (Millipore), and CPSF6 (Novus Biologicals).

HeLa extracts were prepared by seeding 2 million cells in each of 20 100 cm dishes. After culturing for 2 days, cells were detached with trypsin. Cells were pelleted and washed with 5 volumes of cold PBS, and resuspended in 5 volumes of lysis buffer (10 mM Tris-HCl, 1.5 mM MgCl2, 10 mM KCl, 0.5 mM EDTA [pH 8], 1∶100 mammalian protease inhibitor [Sigma]). Lysates were sonicated using a Fisher Scientific Sonic Dismembrator Ultrasonic Processor with the following settings: Amplitude: 7, Process Time: 10 s, Pulse-ON time: 5 s, Pulse-OFF time: 5 s. Sonicated lysates were centrifuged for 30 min at 14,000× g at 4°C to pellet cellular debris. The remaining supernatant was assayed by BCA to determine concentration and aliquots were flash frozen in liquid nitrogen and stored at −80°C. Each aliquot was thawed once prior to use in binding assays.

### Statistical analysis

Differences in infectivity between different conditions (e.g. between control and knockdown, between WT and mutants) were examined by a paired Student *t*-test. *P*-values of 0.05 or less were considered statistically significant.

## Supporting Information

Figure S1 Abrogation of CPSF6-358-mediated restriction by HIV-1 virus particles. HeLa cells expressing CPSF6-358 were inoculated with a fixed dose of WT-crimson virus in the presence of increasing amounts of abrogating GFP virus carrying various CA mutations. One representative data of at least four independent experiments is shown. Compared to viral infectivity without any abrogating virus, infectivity in the presence of 30 µl and 60 µl of WT or T54A virions was increased with statistical significance (p<0.05).
(TIFF)

Figure S2 The N74D mutation significantly reduces association of CPSF6 with CA tubes. (A). The WT, T54A, T54A+A105T, and T54A+N74D CA tubes were incubated with 500, 250, or 125 µg of HeLa cell extracts for one hour with gentle mixing. The tubes were pelleted and analyzed by non-reducing SDS-PAGE. Input represents 10% of each reaction prior to pelleting. (B). Quantification of CPSF6 association with CA tubes relative to amount of pelleted CA. The results are representative of three independent experiments.
(TIFF)

Figure S3 Endogenous CPSF6 restricts cell cycle-dependent CA mutants to varying degrees. VSV-G-pseudotyped GFP reporter viruses were used to infect HeLa cells after siRNA knockdown of CPSF6. The graph was compiled from five independent experiments, while p is *p*-value calculated according to the student's t-test.
(TIFF)

Figure S4 CPSF6 and CypA act together to suppress the cell cycle-dependent capsid mutant T54A. (A) RT-normalized VSV-G-pseudotyped GFP reporter viruses were used to infect HeLa cells in the presence or absence of CsA. T54A was different from all the other viruses in its response to CsA treatment (p<0.05). (B) VSV-G-pseudotyped GFP reporter viruses were used to infect HeLa cells after siRNA knockdown of CPSF6. (C) RT-normalized VSV-G-pseudotyped GFP reporter viruses were used to infect HeLa cells after siRNA knockdown of CypA. Infectivity of only T54A was significantly increased by CPSF6 knockdown (B and C; p<0.02). (D) HeLa cells transfected with siRNA targeting CypA, CPSF6 or both simultaneously were infected with RT-normalized VSV-G-pseudotyped GFP reporter viruses. Results are one representative experiment of at least two experiments. Standard deviations of a single triplicate experiment are indicated with the error bars. (E) CsA does not inhibit CPSF6 association with CA tubes. WT, T54A, T54A+A105T, and T54A+N74D CA tubes (5 µM) were incubated with 125 µg of HeLa cell extracts in the presence or absence of CsA (5 µM) for 1 hour with gentle mixing. The tubes were pelleted and analyzed by non-reducing SDS-PAGE and immunoblotting for CPSF6, CA, and CypA. Input represents 10% of initial reaction. Fold change represents the difference between treated and untreated samples. The results are representative of four independent experiments.
(TIFF)

Figure S5 CPSF6-358 restricts the *in vitro*-derived compensatory mutations S41A and T107I. (A) HeLa cells stably transduced with the control LPCX vector or one overexpressing CPSF6-358 were infected with VSV-G-pseudotyped GFP reporter viruses. Results are one representative experiment of two. Error bars indicate standard deviations of a triplicate experiment. (B) Fold restriction by CPSF6-358 was plotted by compiling all the results of three independent experiments with four different amounts (i.e. twelve independent infections). RKLM+S41A differs from both N74D (p<0.0001) and RKLM+T107I (p<0.0001). RKLM+T107I was statistically different both from WT (p<0.0001) and N74D (p<0.0001).
(TIFF)

Figure S6 Alignment of amino acid sequences of the HIV-1 CA proteins from HLA-B27+ subjects used in this study. HIV-1 capsid sequences from HLA-B27+ subjects, which were previously reported [49] are aligned to that of LAI (K02013). Asterisks indicate no deviation from the LAI sequence.
(TIFF)

Figure S7 Restoration of amino acid residue S41 in *in vivo* variants results in the reacquisition of sensitivity to endogenous RKLM. VSV-G-pseudotyped GFP reporter viruses were used to infect HeLa cells after siRNA knockdown of CPSF6. Results are one representative of three experiments. Error bars indicate standard deviations of a triplicate experiment. Infectivity of CR0206U and CR0339X carrying the reverted serine at 41 (S41) was increased upon CPSF6 depletion (p<0.05).
(TIFF)

Figure S8 Knockdown confirmation by western blot analysis. (A) Western blot of HeLa cells transfected with siRNA targeting CPSF6 or transfection reagent alone were probed with different antibodies (shown in left). (B) Western blot of HeLa cells transfected after siRNA knockdown of CypA were probed with different antibodies (shown in left). (C) Western blot of HeLa cells after siRNA knockdown of TNPO3 or transfection reagent alone were probed with different antibodies (shown in left) (D) Western blot of HeLa cell lysates after infection with VSV-G-pseudotyped crimson reporter viruses carrying shRNA against RanBP2. Either sorted Crimson-positive cells (the second sample from left; lane 2) or the total cells (more than 90% of the cells were crimson-positive) were lysed for western blot and probed with an anti-RanBP2 antibody. (E) Western blot of HeLa cells after siRNA knockdown of Nup153 probed with an anti-nuclear pore complex proteins antibody. (F) Western blot of HeLa cells after siRNA knockdown of specific genes (shown at top) probed with different antibodies (shown in left).
(TIFF)
