# Supplementary figures and images for: In Vivo Functions of CPSF6 for HIV-1 as Revealed by HIV-1 Capsid Evolution in HLA-B27-Positive Subjects
Source: PLoS Pathog. 2014 Jan 9;10(1):e1003868. doi: 10.1371/journal.ppat.1003868 (PMC3887095; doi:10.1371/journal.ppat.1003868)

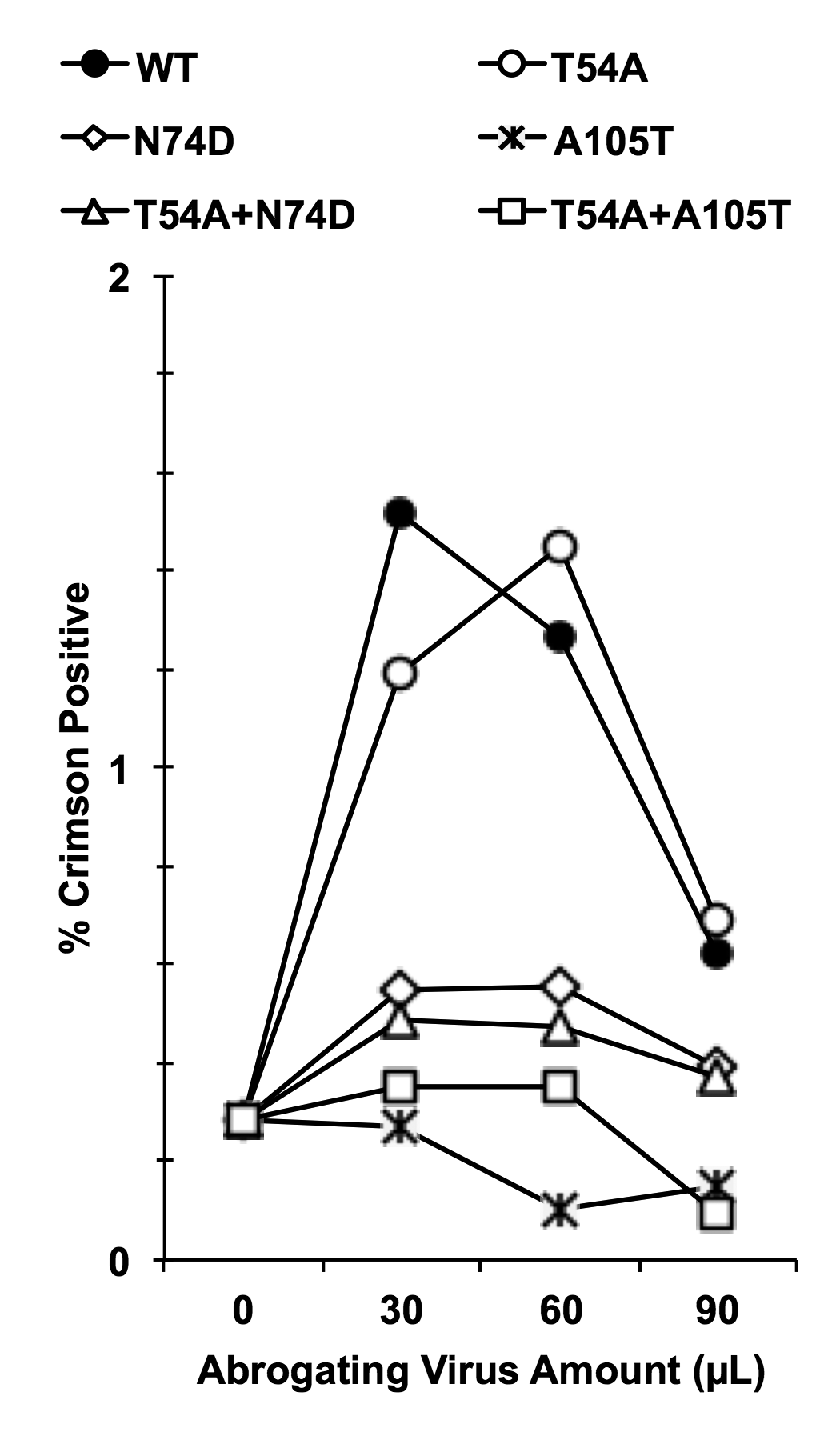

Supplement: Figure S1 — Abrogation of CPSF6-358-mediated restriction by HIV-1 virus particles. HeLa cells expressing CPSF6-358 were inoculated with a fixed dose of WT-crimson virus in the presence of increasing amounts of abrogating GFP virus carrying various CA mutations. One representative data of at least four independent experiments is shown. Compared to viral infectivity without any abrogating virus, infectivity in the presence of 30 µl and 60 µl of WT or T54A virions was increased with statistical significance (p<0.05). (TIFF) [file ppat.1003868.s001.tiff]

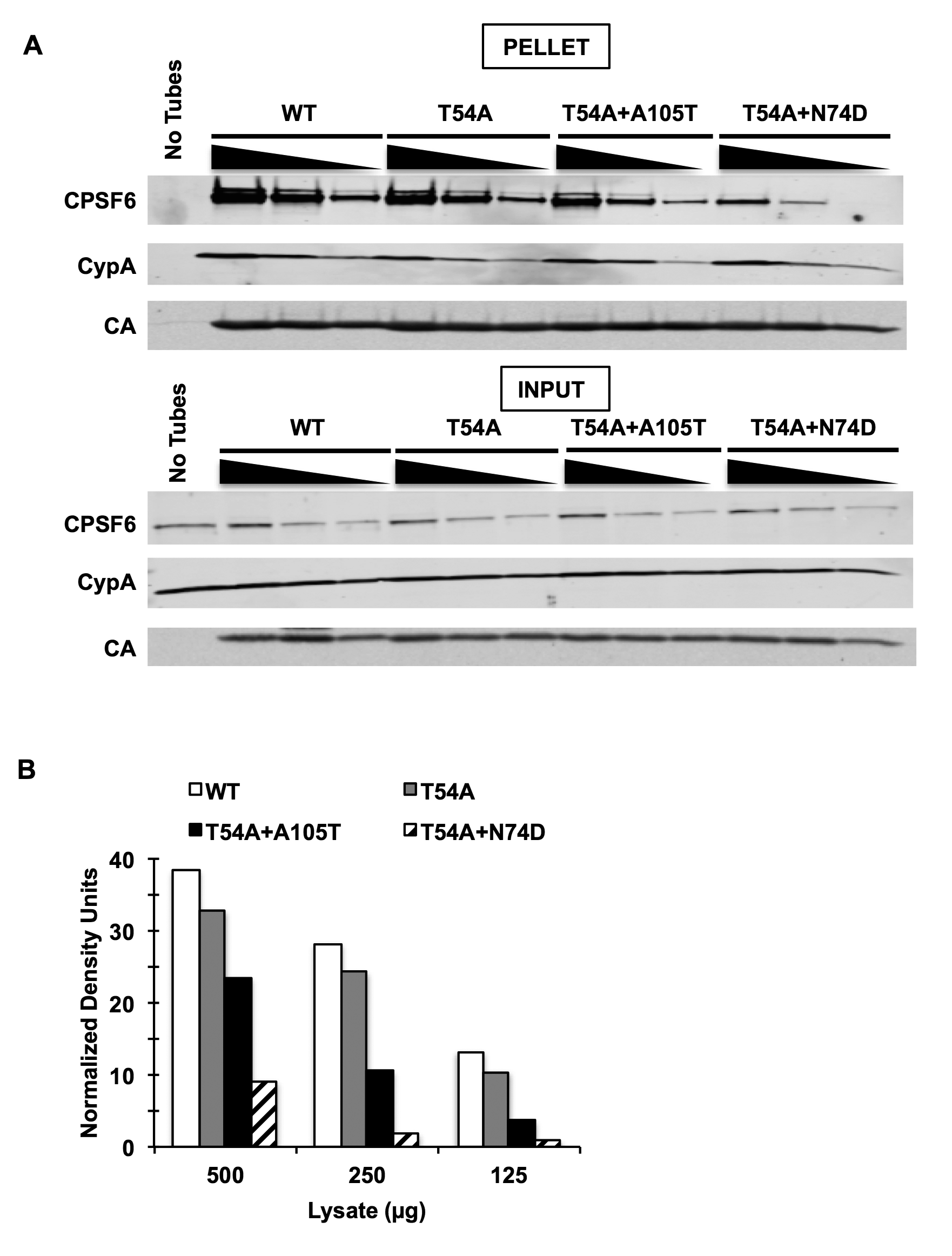

Supplement: Figure S2 — The N74D mutation significantly reduces association of CPSF6 with CA tubes. (A). The WT, T54A, T54A+A105T, and T54A+N74D CA tubes were incubated with 500, 250, or 125 µg of HeLa cell extracts for one hour with gentle mixing. The tubes were pelleted and analyzed by non-reducing SDS-PAGE. Input represents 10% of each reaction prior to pelleting. (B). Quantification of CPSF6 association with CA tubes relative to amount of pelleted CA. The results are representative of three independent experiments. (TIFF) [file ppat.1003868.s002.tiff]

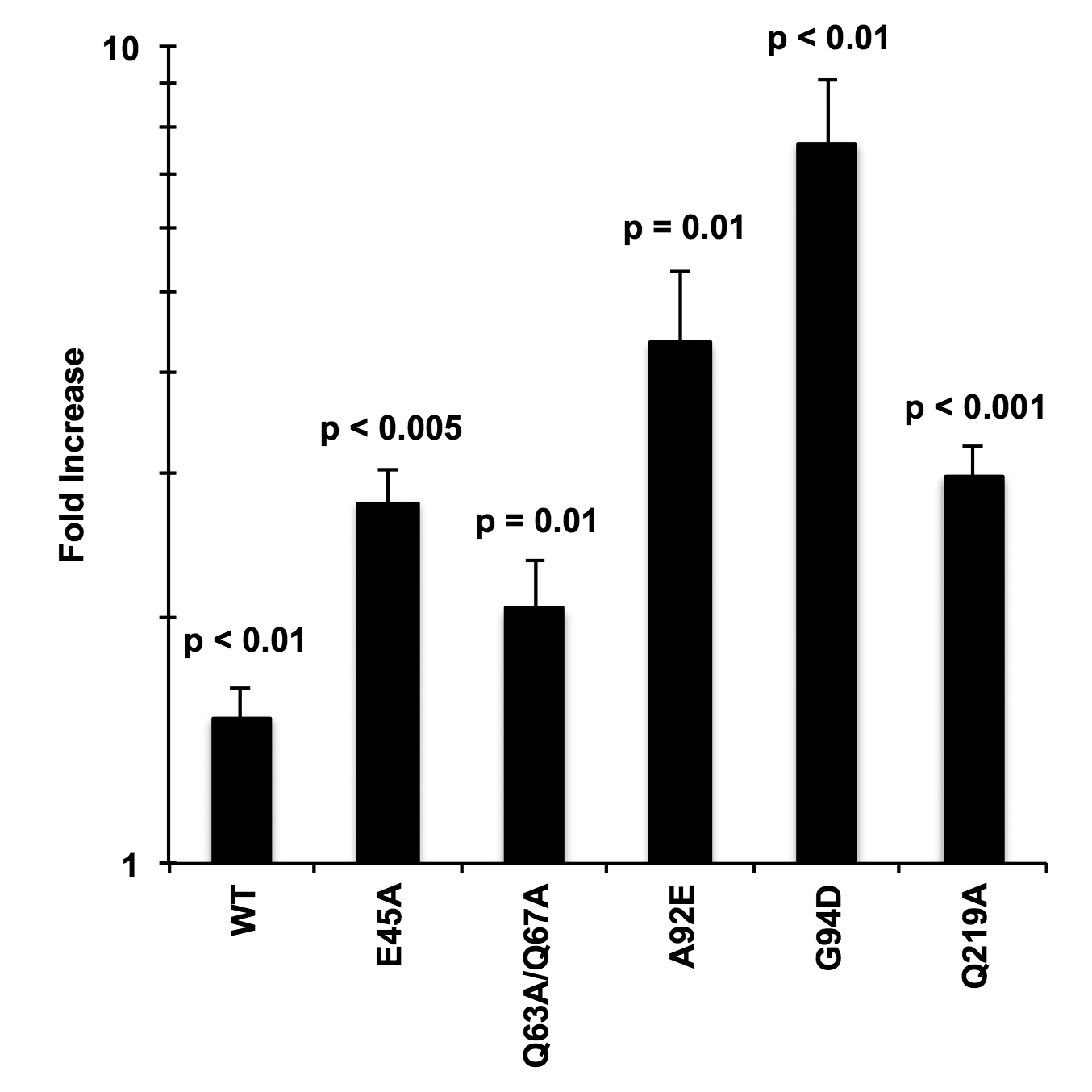

Supplement: Figure S3 — Endogenous CPSF6 restricts cell cycle-dependent CA mutants to varying degrees. VSV-G-pseudotyped GFP reporter viruses were used to infect HeLa cells after siRNA knockdown of CPSF6. The graph was compiled from five independent experiments, while p is p-value calculated according to the student's t-test. (TIFF) [file ppat.1003868.s003.tiff]

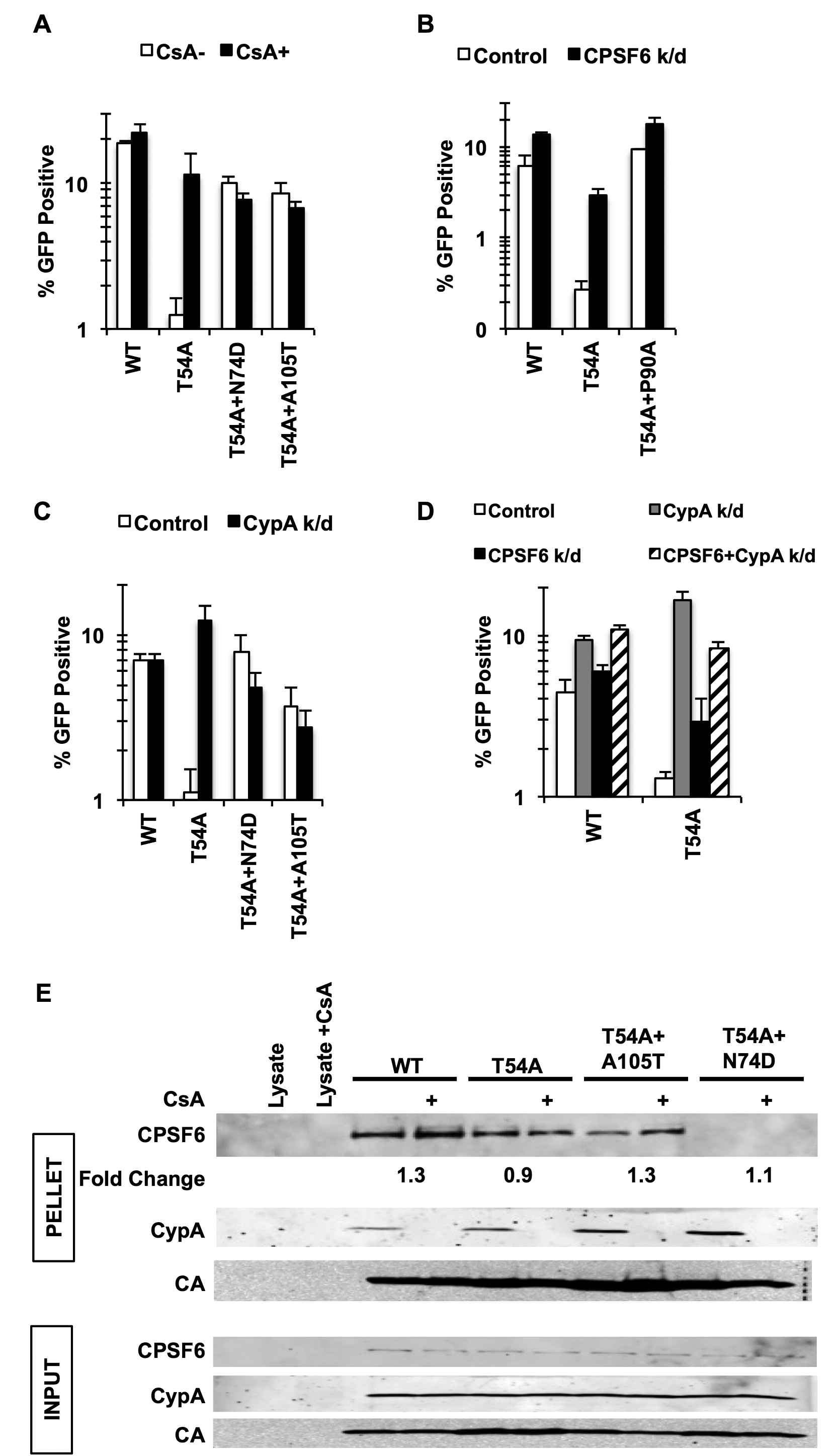

Supplement: Figure S4 — CPSF6 and CypA act together to suppress the cell cycle-dependent capsid mutant T54A. (A) RT-normalized VSV-G-pseudotyped GFP reporter viruses were used to infect HeLa cells in the presence or absence of CsA. T54A was different from all the other viruses in its response to CsA treatment (p<0.05). (B) VSV-G-pseudotyped GFP reporter viruses were used to infect HeLa cells after siRNA knockdown of CPSF6. (C) RT-normalized VSV-G-pseudotyped GFP reporter viruses were used to infect HeLa cells after siRNA knockdown of CypA. Infectivity of only T54A was significantly increased by CPSF6 knockdown (B and C; p<0.02). (D) HeLa cells transfected with siRNA targeting CypA, CPSF6 or both simultaneously were infected with RT-normalized VSV-G-pseudotyped GFP reporter viruses. Results are one representative experiment of at least two experiments. Standard deviations of a single triplicate experiment are indicated with the error bars. (E) CsA does not inhibit CPSF6 association with CA tubes. WT, T54A, T54A+A105T, and T54A+N74D CA tubes (5 µM) were incubated with 125 µg of HeLa cell extracts in the presence or absence of CsA (5 µM) for 1 hour with gentle mixing. The tubes were pelleted and analyzed by non-reducing SDS-PAGE and immunoblotting for CPSF6, CA, and CypA. Input represents 10% of initial reaction. Fold change represents the difference between treated and untreated samples. The results are representative of four independent experiments. (TIFF) [file ppat.1003868.s004.tiff]

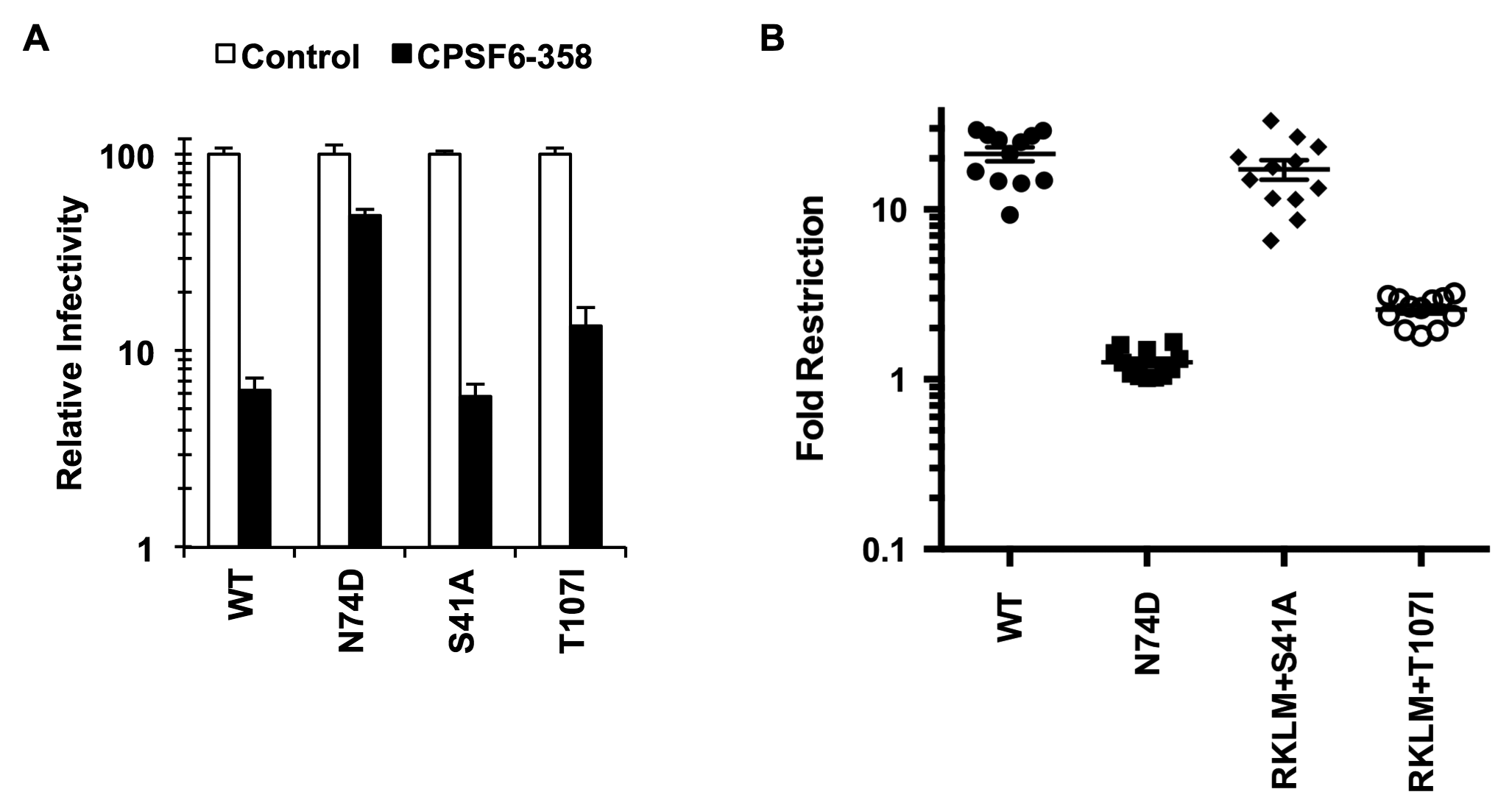

Supplement: Figure S5 — CPSF6-358 restricts the in vitro-derived compensatory mutations S41A and T107I. (A) HeLa cells stably transduced with the control LPCX vector or one overexpressing CPSF6-358 were infected with VSV-G-pseudotyped GFP reporter viruses. Results are one representative experiment of two. Error bars indicate standard deviations of a triplicate experiment. (B) Fold restriction by CPSF6-358 was plotted by compiling all the results of three independent experiments with four different amounts (i.e. twelve independent infections). RKLM+S41A differs from both N74D (p<0.0001) and RKLM+T107I (p<0.0001). RKLM+T107I was statistically different both from WT (p<0.0001) and N74D (p<0.0001). (TIFF) [file ppat.1003868.s005.tiff]

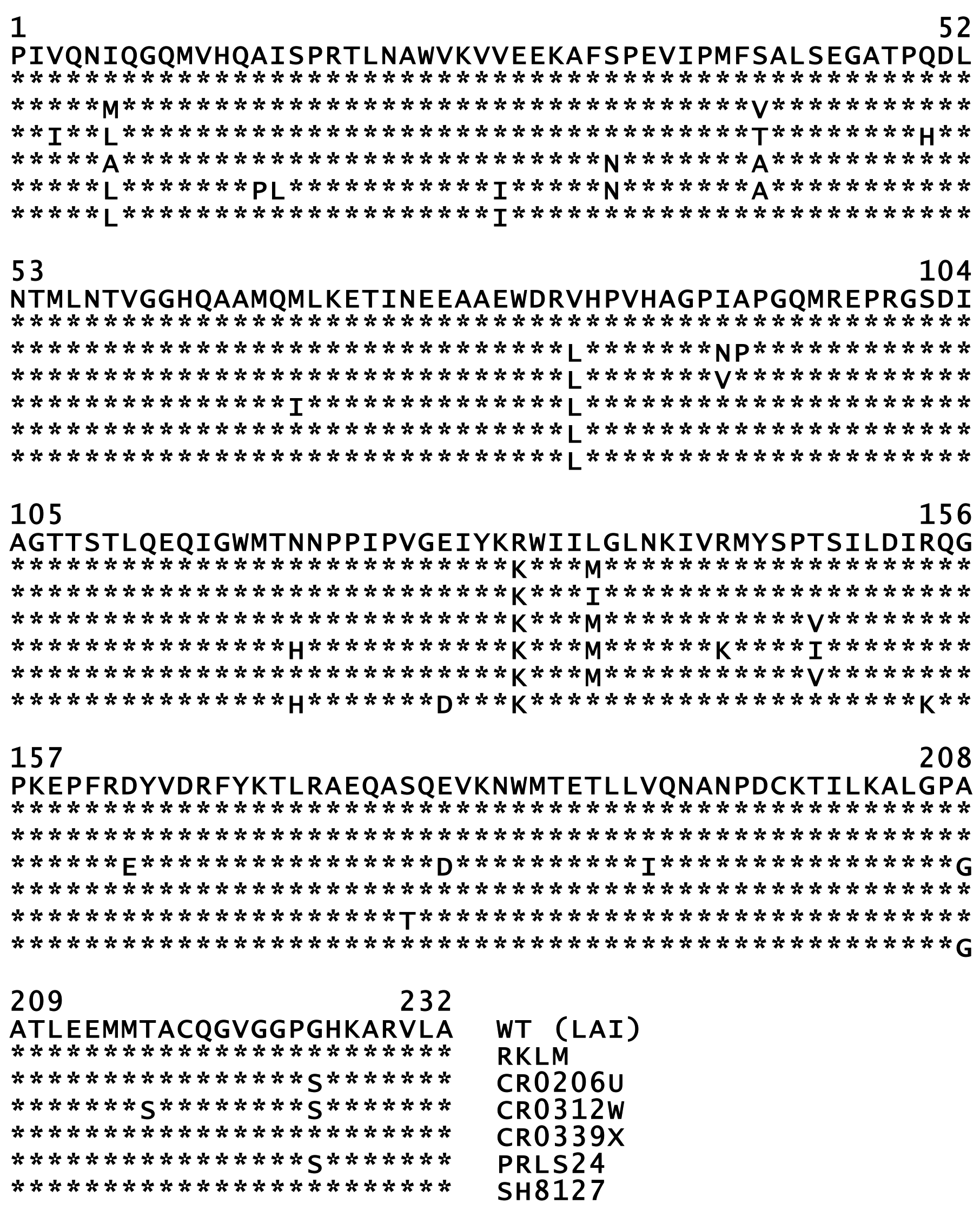

Supplement: Figure S6 — Alignment of amino acid sequences of the HIV-1 CA proteins from HLA-B27+ subjects used in this study. HIV-1 capsid sequences from HLA-B27+ subjects, which were previously reported [49] are aligned to that of LAI (K02013). Asterisks indicate no deviation from the LAI sequence. (TIFF) [file ppat.1003868.s006.tiff]

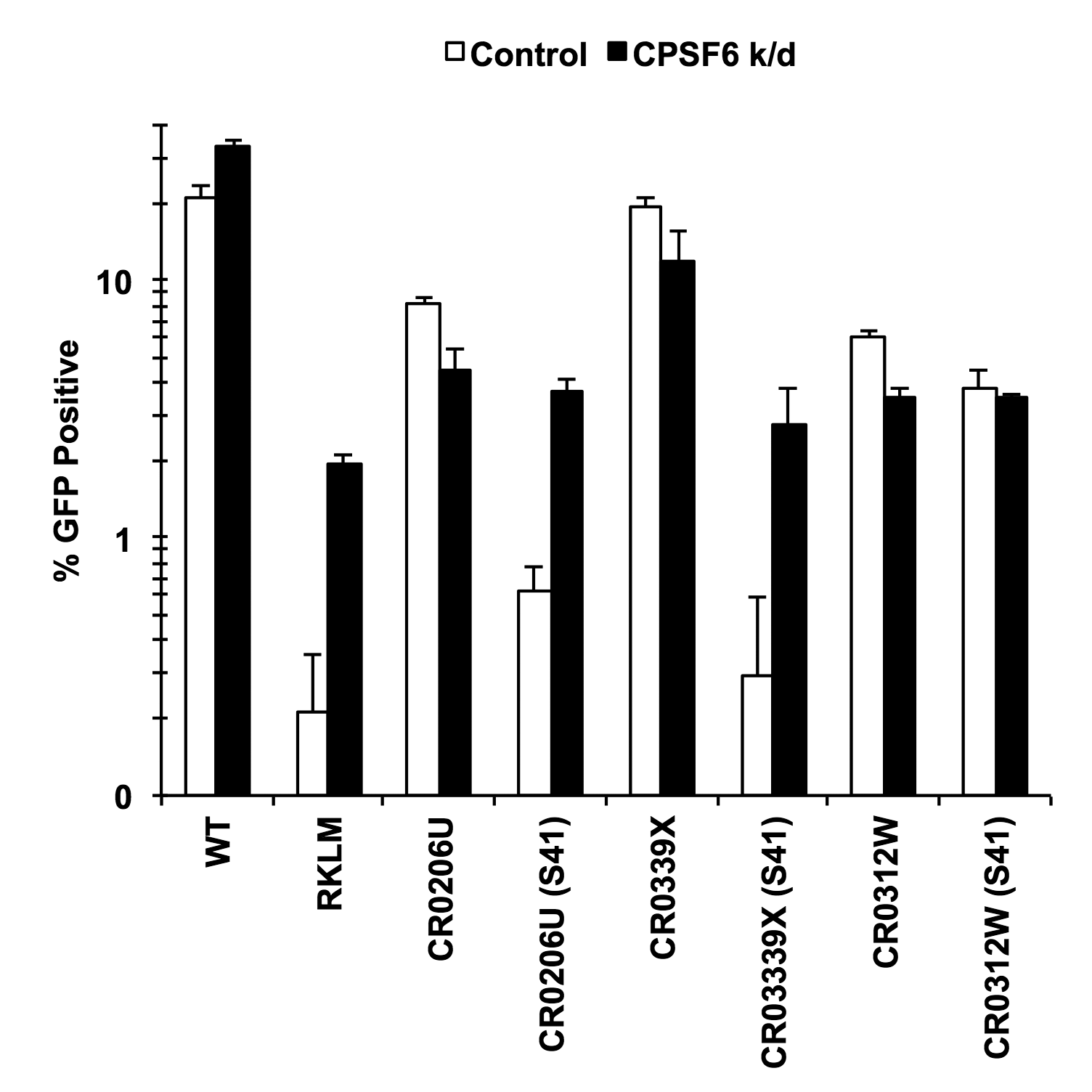

Supplement: Figure S7 — Restoration of amino acid residue S41 in in vivo variants results in the reacquisition of sensitivity to endogenous RKLM. VSV-G-pseudotyped GFP reporter viruses were used to infect HeLa cells after siRNA knockdown of CPSF6. Results are one representative of three experiments. Error bars indicate standard deviations of a triplicate experiment. Infectivity of CR0206U and CR0339X carrying the reverted serine at 41 (S41) was increased upon CPSF6 depletion (p<0.05). (TIFF) [file ppat.1003868.s007.tiff]

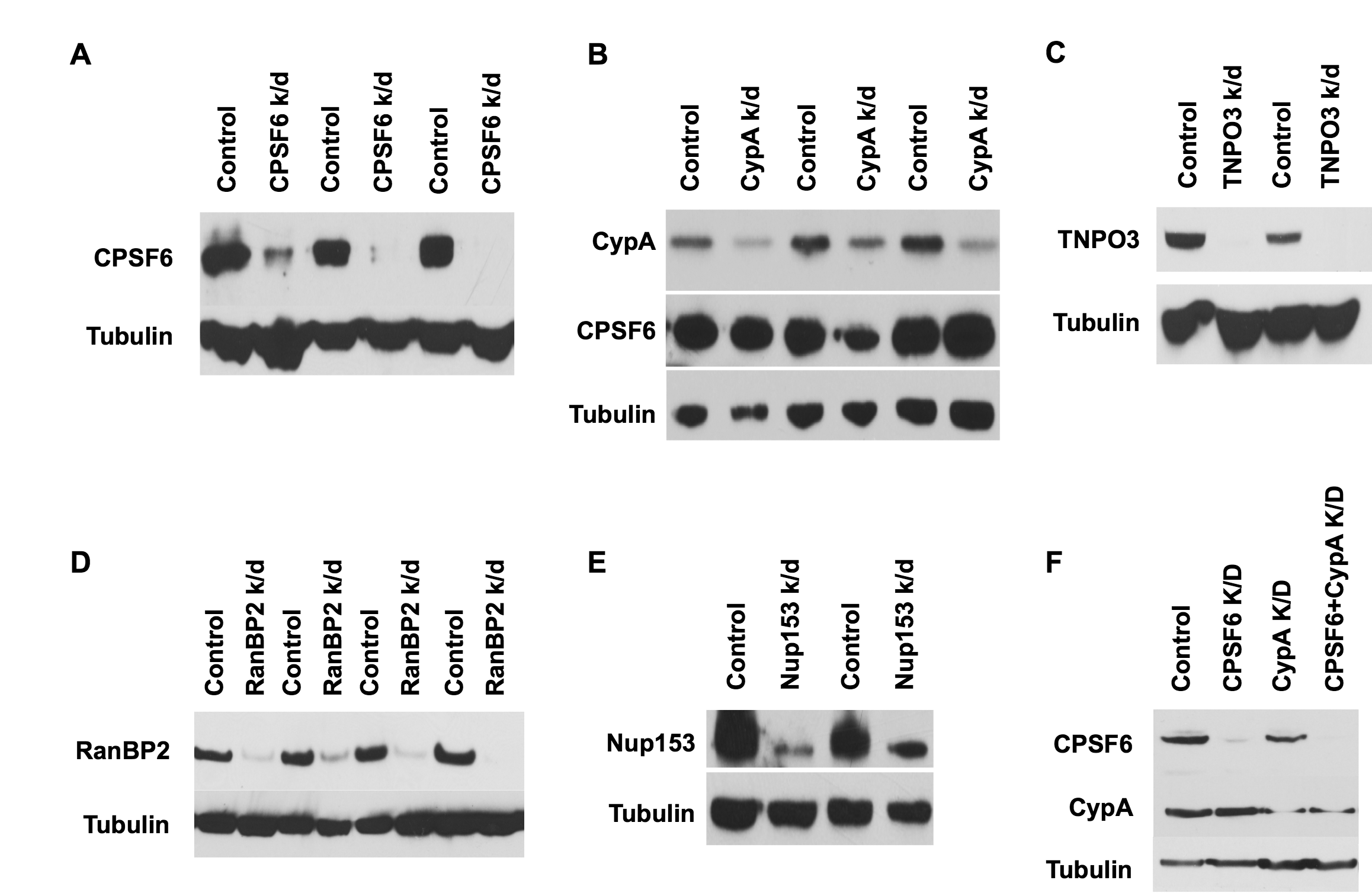

Supplement: Figure S8 — Knockdown confirmation by western blot analysis. (A) Western blot of HeLa cells transfected with siRNA targeting CPSF6 or transfection reagent alone were probed with different antibodies (shown in left). (B) Western blot of HeLa cells transfected after siRNA knockdown of CypA were probed with different antibodies (shown in left). (C) Western blot of HeLa cells after siRNA knockdown of TNPO3 or transfection reagent alone were probed with different antibodies (shown in left) (D) Western blot of HeLa cell lysates after infection with VSV-G-pseudotyped crimson reporter viruses carrying shRNA against RanBP2. Either sorted Crimson-positive cells (the second sample from left; lane 2) or the total cells (more than 90% of the cells were crimson-positive) were lysed for western blot and probed with an anti-RanBP2 antibody. (E) Western blot of HeLa cells after siRNA knockdown of Nup153 probed with an anti-nuclear pore complex proteins antibody. (F) Western blot of HeLa cells after siRNA knockdown of specific genes (shown at top) probed with different antibodies (shown in left). (TIFF) [file ppat.1003868.s008.tiff]
